# Mass Spectrometric Proof of Predicted Peptides: Novel Adipokinetic Hormones in Insects

**DOI:** 10.3390/molecules27196469

**Published:** 2022-10-01

**Authors:** Heather G. Marco, Simone König, Gerd Gäde

**Affiliations:** 1 Department of Biological Sciences, University of Cape Town, Private Bag, Rondebosch, Cape Town ZA-7700, South Africa; 2 IZKF Core Unit Proteomics, Interdisciplinary Center for Clinical Research, University of Münster, Röntgenstr. 21, 48149 Münster, Germany

**Keywords:** neuropeptide, adipokinetic hormone, mass spectrometry, *de novo* sequencing, corpus cardiacum

## Abstract

The importance of insects in our ecosystems is undeniable. The indiscriminate use of broad-spectrum insecticides is a factor in the decline in insect biomass. We identify and sequence a prominent neuropeptide hormone in insects with an overarching goal to elucidate relatedness and create a database of bioactive peptides that could inform possible cross-activity in biological assays for the identification of a biorational lead compound. The major task of an adipokinetic hormone (AKH) in an insect is the regulation of metabolic events, such as carbohydrate and lipid breakdown in storage tissue during intense muscular work. From genomic and/or transcriptomic information one may predict the genes encoding neuropeptides such as the AKHs of insects. Definite elucidation of the primary structure of the mature peptide with putative post-translational modifications needs analytical chemical methods. Here we use high-resolution mass spectrometry coupled with liquid chromatography to identify unequivocally the AKHs of five insect species (one cockroach, two moths, and two flies) of which either genomic/transcriptomic information was available or sequences from related species. We confirm predicted sequences and discover novel AKH sequences, including one with a post-translational hydroxyproline modification. The additional sequences affirm an evolutionary pattern of dipteran AKHs and a conserved pattern in crambid moths.

## 1. Introduction

In insects, as in all other invertebrates and vertebrates, neuropeptides are renowned regulatory substances that control such important processes as homeostasis, development, reproduction, and behavior [1,2]. The neuropeptides are grouped into various families that are characterized by certain primary structural motifs. Some of these families contain highly conserved peptides with only a single form or a few analogs (for example proctolin and crustacean cardioactive peptide), whereas other families have up to 100 known structural analogs. This is, for example, the case with the so-called adipokinetic hormone (AKH)/red pigment-concentrating hormone (RPCH) family [3,4]. Because the AKH/RPCH peptides display pleiotropic actions, in this publication we use the generic name AKH and direct the interested reader to reviews where the relationship between AKH and the superfamily of gonadotropin-releasing hormone is widely discussed [4,5,6]. In insects, AKHs are almost exclusively synthesized in neurosecretory cells of the retrocerebral corpus cardiacum (CC) gland. The genes of many AKHs are known; the first one was published from the sphingid moth *Manduca sexta* [7], which showed the general organization of the AKH precursor: a signal peptide is followed by the encoded AKH, a glycine amidation site, a dibasic splicing site, and a second peptide referred to as the AKH-precursor-related peptide of which no function is known.

The biosynthetic pathway of AKHs in locusts is well researched and reviewed [8,9], with reports of the unique formation of dimeric prohormones before cleavage to the mature AKHs. In general, the mature AKHs of insects are chemically characterized by the following: they are 8 to 10 amino acids long, have blocked N- (pyroglutamate) and C- (carboxyamide) termini, aromatic amino acids at position 4 (phenylalanine or tryptophan) and 8 (tryptophan), and a glycine residue at position 9 (see, for example [10,11]). Besides the post-translational modifications at the termini, the following modifications have been reported: a very unusual C-mannosylation at the tryptophan residue [12,13], a tryptophan replaced with kynurenine [14], phosphorylation [15], hydroxyprolination [16], sulfation [17] and, lastly, a possible cis-trans proline isomerization [18].

Following the release of an AKH into the hemolymph, it binds to a G protein-coupled receptor and activates either a triacylglycerol lipase or a glycogen phosphorylase in the fat body to elicit a hyperlipemic (adipokinetic) or a hypertrehalosemic response, respectively [19]. Thus, AKHs are mainly metabolic hormones in insects, such as glucagon is in vertebrates, making fuels available during high-energy demanding periods in insects like flight activity (for more information see, for example [4,20]).

Twenty years ago, the first insect genome was published from the vinegar fly *Drosophila melanogaster* [21]. Since then, many more insect genomes have been unraveled, the majority from holometabolous insects. Currently, the i5k project (http://i5k.github.io/, accessed on 29 September 2022), which pledged to sequence the genomes of 5000 arthropod species, provides new data on a regular basis. There is also an ever-increasing number of transcriptomes (ESTs, expressed sequence tags) available, which can be mined (see, for example, [22,23]). This genomic/transcriptomic information can be used for the prediction of genes encoding neuropeptides such as the AKHs. Bioinformatic predictions, however, are not sufficient to elucidate the structure of a mature AKH. Post-translational modifications are not encoded and the presence of a gene is not a given fact of expression in the organism. Thus, to arrive at definitive information on the primary sequence of an AKH in any given species, it is imperative to isolate the peptide from the insect and validate its sequence using analytical chemistry methods. In the current study, we use high-resolution mass spectrometry (MS) coupled with nanoflow reversed-phase liquid chromatography (LC) to investigate AKHs from five insect species, of which genomic/transcriptomic information was available from three of the species to supply predictive sequence information. The insects comprise hemimetabolous (a termite of the order Blattodea) and holometabolous insects (two species each of fly and moth belonging to the orders Diptera and Lepidoptera, respectively). None of these species were previously analyzed for the chemical structure and complement of AKHs.

## 2. Results

**Figure 1:**
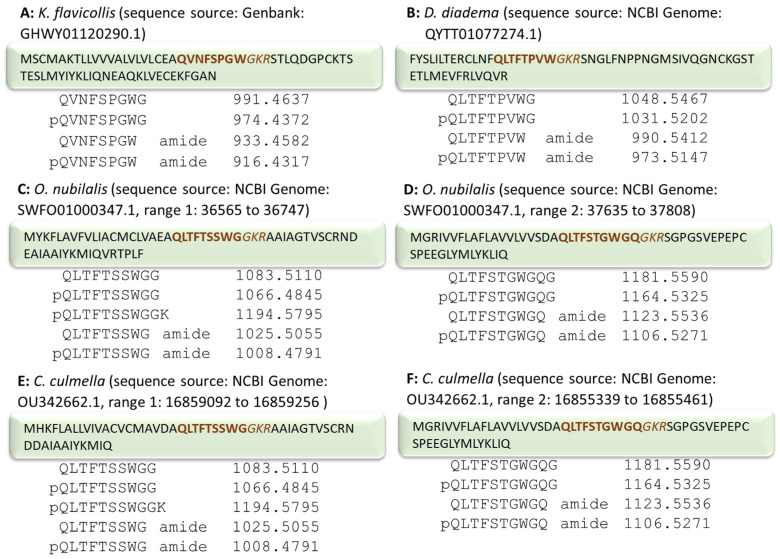
Amino acid sequences for putative mature AKHs deduced from precursor sequences mined from various databases. The sequence of the encoded potential AKH is given in **bold letters** and the possible amidation site plus dibasic cleavage site in *italics*. The calculated protonated mass for potentially processed peptide is shown. The insects are *Kalotermes flavicollis* (**A**), *Dasypogon diadema* (**B**), *Ostrinia nubilalis* (**C**,**D**), and *Chrysoteuchia culmella* (**E**,**F**).

We studied CC extracts of the termite *Kalotermes flavicollis*, family Kalotermitidae, the robber fly *Pegesimallus tapulus*, family Asilidae, the horse fly *Haematopota pluvialis*, family Tabanidae, the European maize borer *Ostrinia nubilalis*, family Crambidae, and the garden grass-veneer *Chrysoteuchia culmella*, family Crambidae. Genomic information from *O. nubilalis* and *C. culmella* was available for data mining via the Genome Browse function in the National Center for Biotechnology Information (NCBI) database. Transcriptome shotgun assembly was also accessible in NCBI from *K. flavicollis*. AKH peptide precursor prediction (Figure 1) was accomplished using the BLAST procedure. Each deduced protein was assessed for typical peptide precursor features, such as a full-length open reading frame, the presence of a signal peptide sequence and pro-hormone convertase cleavage sites, and homology to known AKH peptide isoforms (post-translational modifications, e.g., cyclization of amino (N)-terminal Gln/Glu residues and carboxyl (C)-terminal amidation at Gly residues were predicted by homology). From these predicted sequences, mass data could be calculated and used in MS-targeted analyses with crude CC extracts. For *P. tapulus* the genome of another robber fly species, viz. *Dasypogon diadema* was mined (Figure 1), whereas the published AKH sequences of the tabanid horse fly *Tabanus atratus* [24] were used as a predictive guide in the MS screens of AKHs in *H. pluvialis*. Additionally, previously published dipteran AKH sequence data [25] were employed as potential targets in analyzing the CC extracts from the robber fly and common horse fly in the current study. Peptide assignments were validated by applying a synthetic standard of the proposed primary structure to the same instrumentation and under the same physical conditions as for the native CC extract.

### 2.1. AKH of the Termite Kalotermes flavicollis, Family Kalotermitidae

**Figure 2:**
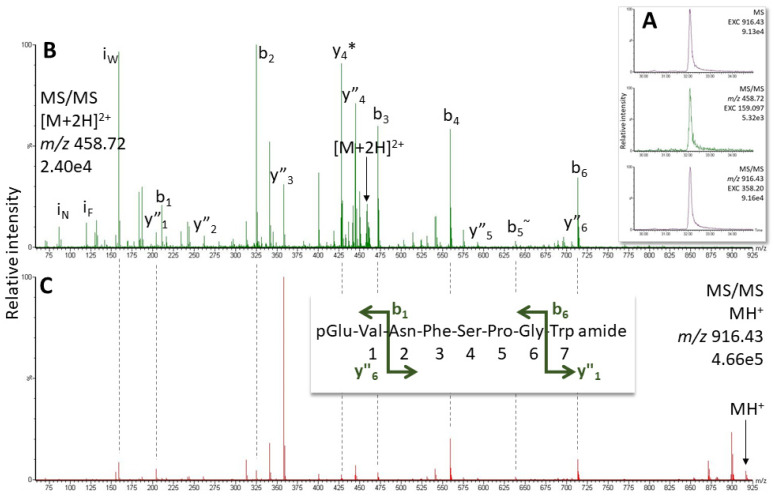
MS target analysis for Manto-CC in *K. flavicollis* (0.5 µL injection, equivalent to the glandular content of 0.2 CC). (**A**) Extracted ion chromatograms (EXC), from the top: for the singly-charged peptide ion (*m*/*z* 916.43) in the overview scan; for the Trp immonium ion in the MS/MS scan of the doubly-charged peptide ion (*m*/*z* 458.72); and for the MS/MS of the singly-charged peptide ion (*m*/*z* 916.43) on the dominant y_3_ ion. (**B**,**C**) MS/MS spectra of the singly- (**C**) and the doubly- (**B**) charged peptide ions. Peaks were labeled according to the b- and y-ion series as calculated in Supplementary Figure S1. For original spectra, also see Figure S1; for validation see Figure S2.

From the assembled transcriptome in NCBI, an AKH precursor can be predicted and a potential AKH is deduced based on sequence homologies (Figure 1A). However, differential processing of this precursor could potentially lead to various isoforms of the AKH peptide, as indicated in Figure 1A. When an aliquot of 0.2 CC gland extract from *K. flavicollis* was separated and analyzed using LC-MS, the ion with a retention time (RT) of 32.1 min (Figure 2A) was tentatively identified as Manto-CC (pQVNFSPGWa) by target analysis on the singly- and doubly-charged ions (Figure 2). The measured *m*/*z* value in the overview scan corresponded to the expected *m*/*z* for protonated Manto-CC (916.432) and the gas phase fragmentation of the singly- and doubly-charged peptide ions confirmed the assignment (Figure 2B,C and Supplementary Figure S1). The assignment was validated by running the synthetic standard, which showed an identical MS/MS spectrum (Supplementary Figure S2). Masses that correspond to potential alternatively processed forms of Manto-CC were not observed.

### 2.2. AKHs of Flies (Diptera)

#### 2.2.1. Robber Fly *Pegesimallus tapulus*, Family Asilidae

**Figure 3:**
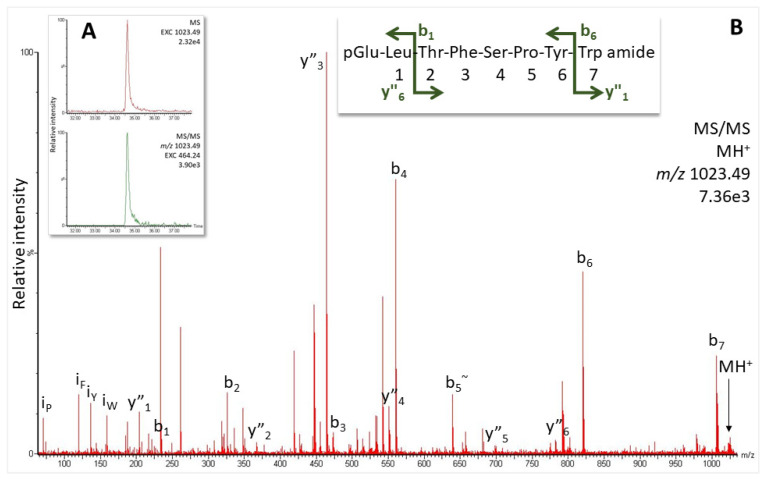
MS target analysis for Volpe-CC in *P. tapulus* (1 µL injection, equivalent to the glandular content of 0.85 CC). (**A**) Extracted ion chromatograms (EXC), from the top: for the singly-charged peptide ion (*m*/*z* 1023.49) in the overview scan and for the MS/MS of the singly-charged peptide ion on the dominant y_3_ ion. (**B**) MS/MS spectrum of the singly-charged peptide ion. Peaks were labeled according to the b- and y-ion series as calculated in Supplementary Figure S3. For original spectrum also see Figure S3; for validation, see Figure S4.

The only information available to date on AKHs in the dipteran family of Asilidae is a precursor mined from a shotgun whole genome of the species *Dasypogon diadema* (Figure 1B; NCBI accession number QYTT01077274.1). The putative mature peptide pQLTFTPVWa could be deduced from the precursor as a potential novel mature AKH in *D. diadema* [25] but could not be confirmed due to the unavailability of specimens, hence the lack of glandular material. In the current study, we were able to collect the CCs from a robber fly from South Africa, *P. tapulus*. An overview MS scan did not reveal any of the masses that could relate to an AKH identical to that predicted for *D. diadema* (Figure 1B), instead, a mass (*m*/*z* 1023.49) that indicated the known structure of Volpe-CC (pQLTFSPYWa, [25]) was detected. Subsequent target MS/MS analysis in the methanolic extract showed a response at 34.6 min (Figure 3A) with an MS/MS matching the ions expected for this sequence (Figure 3B and Supplementary Figure S3). The assignment was confirmed with the corresponding synthetic peptide (Supplementary Figure S4).

**Figure 4:**
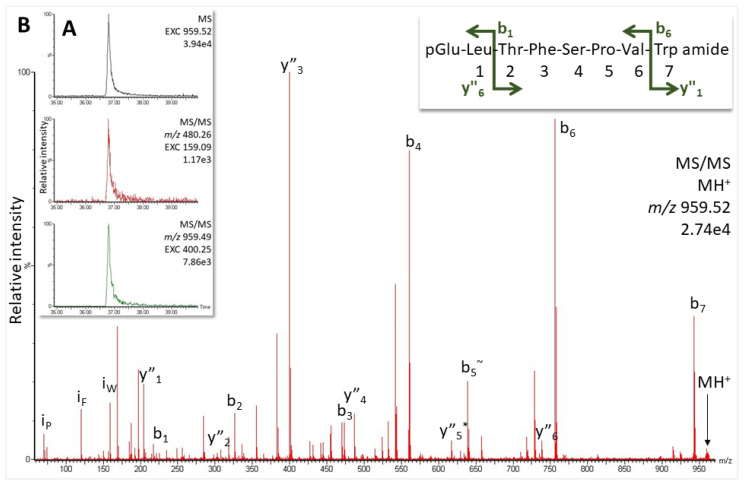
MS screening for potential AKHs in *P. tapulus* (1 µL injection, equivalent to the glandular content of 0.85 CC). (**A**) Extracted ion chromatograms (EXC), from the top: for the singly-charged peptide ion (*m*/*z* 959.52) in the overview scan; for the Trp immonium ion in the MS/MS scan of the doubly-charged peptide ion (*m*/*z* 480.26); and for the MS/MS of the singly-charged peptide ion on the dominant y_3_ ion. (**B**) MS/MS spectrum of the singly-charged peptide ion. Peaks were labeled according to the b- and y-ion series as calculated in Supplementary Figure S5 for sequence pQLTFSPVWa. For original spectrum also see Figure S5; for validation, see Figure S6.

Furthermore, screening for more AKHs using the Trp immonium ion as a marker in data-independent measurements with increased collision energy revealed another potential neuropeptide at 36.8 min and *m*/*z* 959.52 (Figure 4A) that was, hitherto, not associated with a known dipteran AKH (see Table 1 in [25]). Analysis of the MS/MS data revealed the sequence pQLTFSPVWa (Figure 4B and Supplementary Figure S5), a novel peptide which we now code-name Pegta-AKH. The use of the synthetic standard in our MS analyses confirmed the novel sequence information (Supplementary Figure S6).

#### 2.2.2. Horse Fly *Haematopota pluvialis*, Family Tabanidae

**Figure 5:**
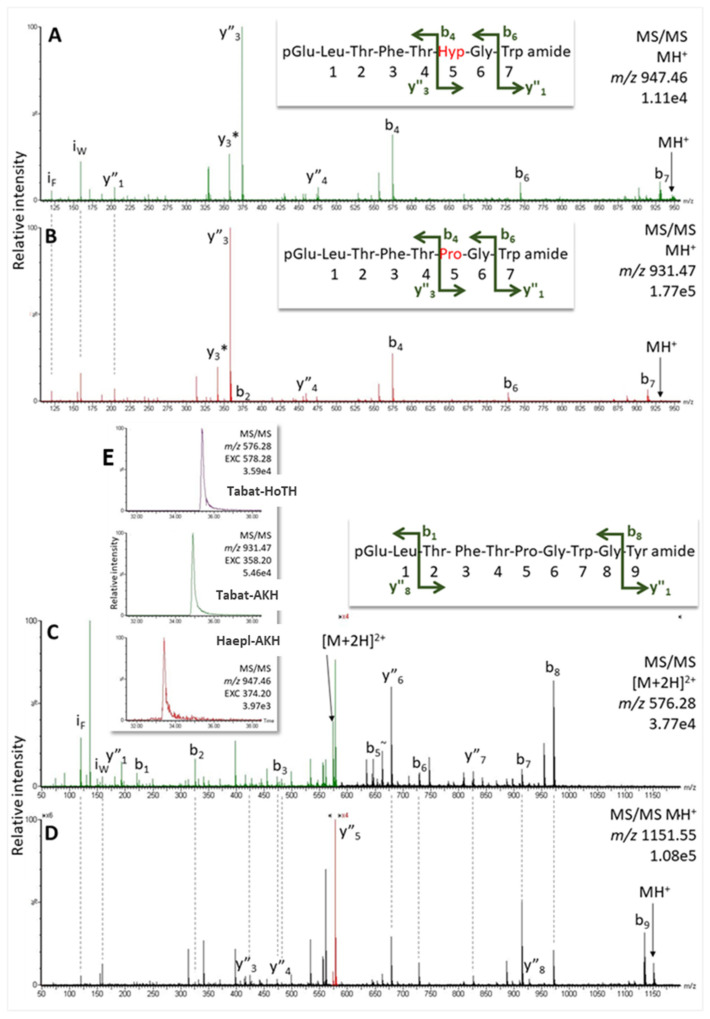
MS analysis for AKHs in *H. pluvialis* (1 µL injection, equivalent to the glandular content of 0.55 CC). (**A**,**B**,**D**) MS/MS spectra of the singly-charged peptide ions of (**A**) a novel peptide (Haepl-AKH)—a hydroxyproline form of Tabat-AKH; (**B**) Tabat-AKH, and (**D**) Tabat-HoTH. Peaks were labeled according to the b- and y-ion series as calculated in the original spectra (Supplementary Figures S7 and S9; validated in Supplementary Figures S8, S10 and S11). (**C**) MS/MS of doubly-charged ion of Tabat-HoTH. (**E**) Extracted ion chromatograms (EXC) from the top: for the dominant y_5_ fragment ion (*m*/*z* 578.28) in the MS/MS of the doubly-charged peptide ion (*m*/*z* 576.28) for Tabat-HoTH; for the dominant y_3_ fragment ion (*m*/*z* 358.20) in the MS/MS of the singly-charged peptide ion (*m*/*z* 931.47) for Tabat-AKH, and for the dominant y_3_ ion (*m*/*z* 374.20) in the MS/MS of the singly-charged peptide ion (*m*/*z* 947.46) for Haepl-AKH.

We collected gland material from the common horse fly *H. pluvialis* of which no genomic/transcriptomic information was available. However, the AKH sequences of another horse fly species, *Tabanus atratus*, had been reported previously (Tabat-AKH: pQLTFTPGWa, Tabat-HoTH: pQLTFTPGWGYa, [24]); as a first guide, we looked for masses that corresponded to these peptides in the CC extracts of *H. pluvialis*. An overview mass screening of the CC extract, indeed, revealed masses that matched to these two peptides (Figure 5E). A signal at 34.9 min (*m*/*z* 931.47) presented MS/MS data as expected for Tabat-AKH (Figure 5B and Supplementary Figure S9), whereas the mass signal at 35.4 min (*m*/*z* 1151.55) showed gas phase fragmentation like Tabat-HoTH (Figure 5D and Supplementary Figure S7). The assigned amino acid sequences were validated in MS with corresponding synthetic peptides (see Supplementary Figures S8 and S10).

Further screening of the sample revealed a potential third AKH candidate in *H. pluvialis* CC: at 33.4 min RT with 16 Da higher than Tabat-AKH (i.e., *m*/*z* 947.46; Figure 5E). The resulting MS/MS spectrum resembled that of Tabat-AKH in its general ion abundancies, but dominant ions such as y_3_ and b_6_ differed also by 16 Da, whereas b_4_ appeared at the same *m*/*z* value (see Figure 5A,B). This observation indicated oxidation of the Pro residue in position 6. Exact mass measurement of the parent ion peak further supported this hypothesis: the singly-charged ion of hydroxyproline-containing Tabat-AKH (i.e., Haepl-AKH) was expected at *m*/*z* 947.46 and this matched the actual peak location. Comparison with the synthetic standard confirmed the postulated sequence of this novel peptide in *H. pluvialis*, as shown in Supplementary Figure S11.

### 2.3. AKHs of Moths (Lepidoptera)

#### 2.3.1. European Corn Borer *Ostrinia nubilalis*, Family Crambidae

**Figure 6:**
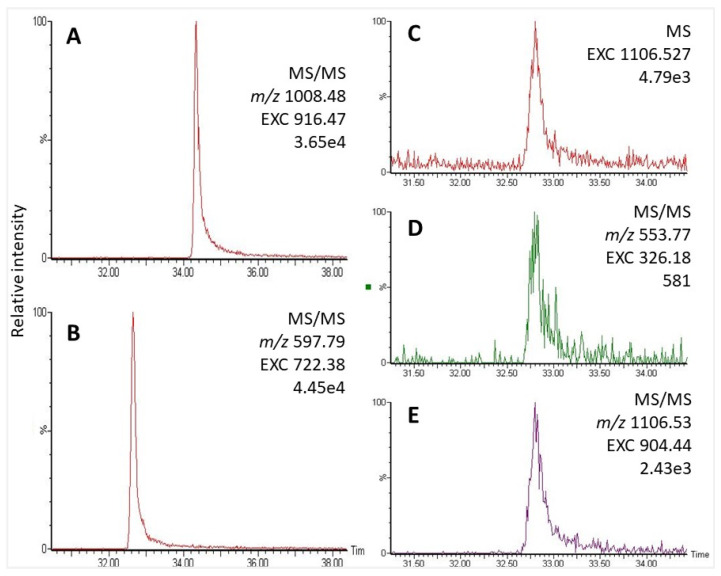
Chromatograms for signals in the methanolic CC extract from the corn borer *O. nubilalis* assigned to (**A**) Manse-AKH (EXC b_7~_), (**B**) Vanca-AKH (EXC y_7_), (**C**–**E**) Ostnu-AKH: (**C**) MS of the parent ion; (**D**) MS/MS doubly-charged ion, EXC b_2_; (**E**) MS/MS singly-charged ion, EXC b_7_.

**Figure 7:**
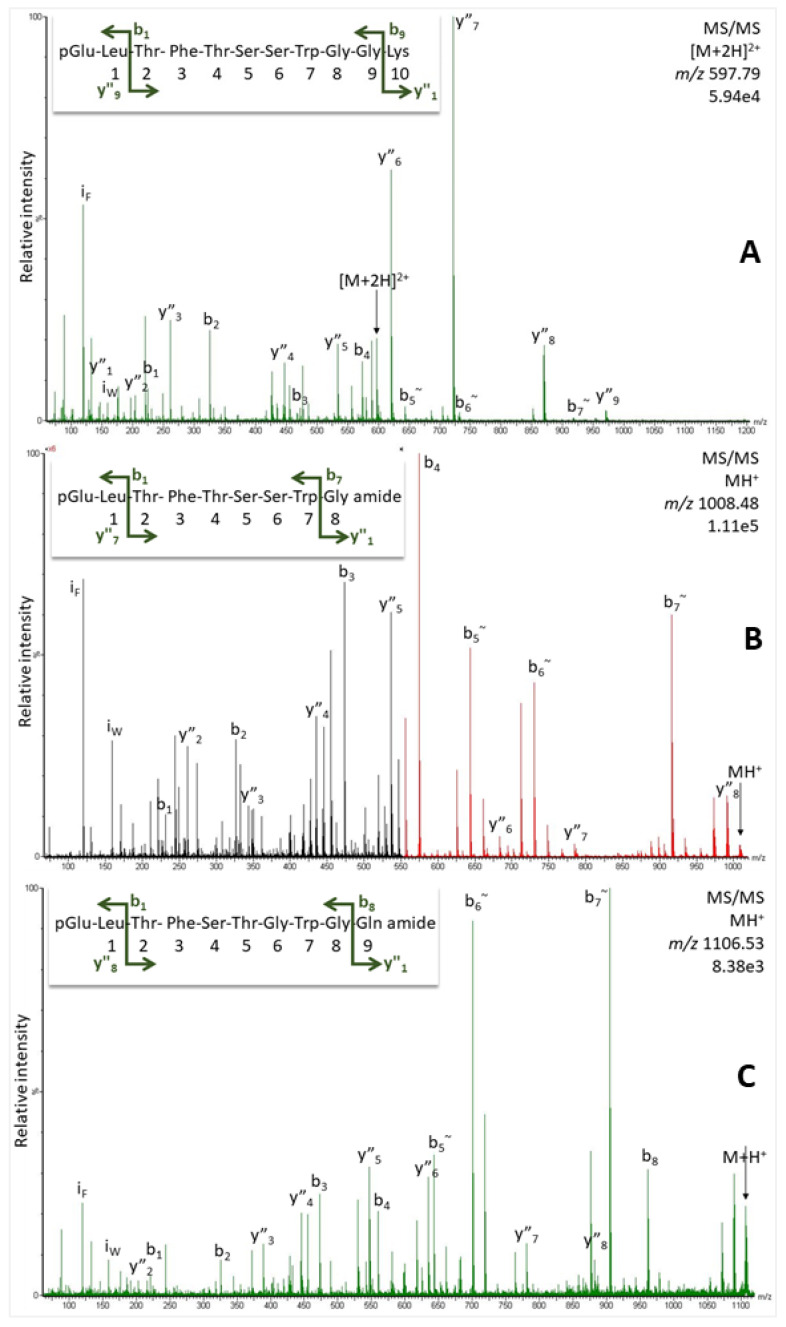
MS/MS spectra for peptides in *O. nubilalis* assigned to (**A**) Vanca-AKH, (**B**) Manse-AKH, and (**C**) Ostnu-AKH of the doubly- (**A**) and singly-charged peptide ions (**B**,**C**): 1 µL injection, equivalent to the glandular content of 1.25 CC. Peaks were labeled according to the b- and y-ion series as calculated in the original spectra in Supplementary Figures S12, S13 and S15. For sequence validation, see Supplementary Figures S14 and S16.

Two precursors of AKHs could be mined from the NCBI genomic database for the European corn borer *O. nubilalis* (Figure 1C,D). In addition, previously published lepidopteran AKH sequence and mass data [26] were employed as a guide. In Figure 1C,D we also indicate the putative mature peptides that may be derived from the precursors. From our mass spectrometric studies using a methanolic extract from the CC of *O. nubilalis*, signals corresponding to the predicted peptides were observed at *m*/*z* 1008.48 (34.3 min) and *m*/*z* 597.79 (32.6 min, Figure 6A,B) that correlate with the known peptide structures Manse-AKH (pQLTFTSSWGa) and Vanca-AKH (pQLTFTSSWGGK), respectively [26]. Gas phase fragmentation of these peptides confirmed the amino acid assignment (Figure 7A,B and Supplementary Figures S12 and S13), and it was further validated with the corresponding synthetic peptide (Supplementary Figure S14).

In addition, at 32.8 min a signal (*m*/*z* 1106.53) was seen at about a tenth of the intensity of the other two AKHs (Figure 6C–E), which could be matched to the predicted amino acid sequence, pQLTFSTGWGQa (Figure 1D). The MS/MS spectrum of this peptide (Figure 7C and Supplementary Figure S15) as well as the validation with the synthetic standard (Supplementary Figure S16) support the sequence assignment, and we code-name this novel peptide Ostnu-AKH as this is the first time of its chemical sequencing.

#### 2.3.2. Garden Grass-Veneer *Chrysoteuchia culmella*, Family Crambidae

**Figure 8:**
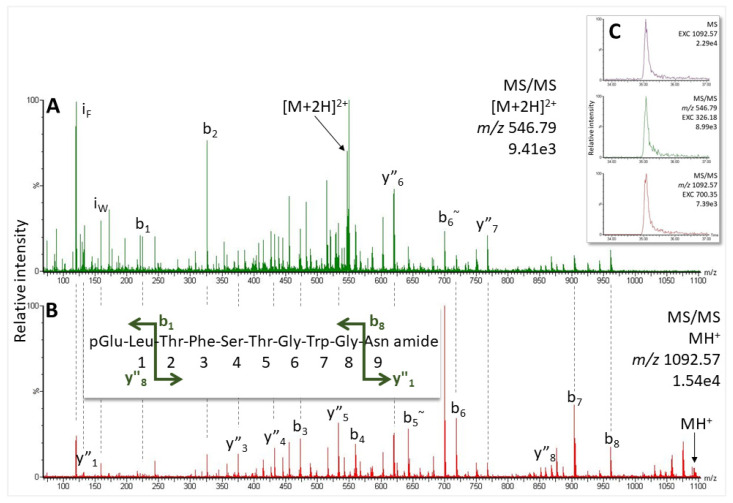
MS/MS spectra for the peptide assigned to Chipa-AKH in *C. culmella* of the (**A**) doubly- and (**B**) singly-charged ions (1 µL injection, equivalent to the glandular content of 0.65 CC). Peaks were labeled according to the b- and y-ion series as calculated in Supplementary Figure S19. For original spectra also see Figure S19 and for validation Figure S20. (**C**) EXC, from the top: for singly-charged ion at *m*/*z* 1092.57 in the MS scan; for b_2_ in the MS/MS of the doubly-charged ion; and for b_6_~ in the MS/MS of the singly-charged ion.

A genome assembly in NCBI Genome Browser is available for this species. Two putative AKH preprohormones were identified from the genome from which we could predict putative mature AKHs alongside their respective mass data (Figure 1E,F). In the CC methanolic extract from *C. culmella* we detected mass signals that did correspond to Manse-AKH (*m*/*z* 1008.48), and also Vanca-AKH (*m*/*z* 1194.58; see Supplementary Figure S17 and for validation, Figure S18). Furthermore, a third predicted peptide was measured at 35.0 min, which could be matched to an already-known structure (Chipa-AKH [26]) based on its MS/MS data (Figure 8 and Supplementary Figure S19) and the comparison with the spectrum of the synthetic peptide (Supplementary Figure S20).

## 3. Discussion

The main objective of the current research was to identify mature AKHs by chemical methodologies in those species of which genomic/transcriptomic information was known. As the biosynthesis of these neuropeptides follows general principles and produces first a large precursor protein, it is not clear from such information whether in all cases (1) the glutamine residue is modified to pyroglutamate, (2) a glycine residue is modified to a carboxyamide, and/or (3) the dibasic splicing site is cleaved differently and a partially processed AKH is formed (see Figure 1), or whether additional post-translational modifications may further alter the AKH peptide.

The starting material for this study was an extract from the microscopic neuroendocrine gland, the corpora cardiaca (CC), from specific insect species. The CC synthesizes and stores several other neuropeptides besides the AKHs [2], as can be seen from the overview of LC-MS profiles in Supplementary Figure S21. In our experimental set-up, the AKHs do not dominate the LC-MS runs and we have, therefore, used different MS-based approaches to detect the predicted AKH peptides in the insect crude extracts and to validate sequence information.

Our data set reveals clearly that in all cases, a pyroglutamate is formed at the N-terminus and an amidation at the C-terminus of an AKH. Only in the lepidopteran species do we find, *inter alia*, a peptide, which is unconventionally cleaved from the precursor; it is elongated by glycine and lysine and is not amidated. Such an incompletely processed undecapeptidic AKH was found previously [26,27,28] and was code-named Vanca-AKH, but stems from the same precursor as the nonapeptide Manse-AKH which is also found in *O. nubilalis* and in *C. culmella* (Table 1).

More importantly, a second AKH, which was predicted from one of the precursors of *O. nubilalis* (Figure 1) has also been confirmed unequivocally to occur in the CC extract (Figure 7C). It is a novel decapeptide, now code-named Ostnu-AKH (Table 1), that is also predicted to co-occur with Manse-AKH in another *Ostrinia* species, viz. *O. furnacalis* [26] (NCBI accession no. XP_028164238 and XP_028164252.1). With a glutamine residue at position 10, Ostnu-AKH is closely related to the peptide Chipa-AKH (an asparagine residue at position 10) that was previously sequenced in the crambid moth *Chilo partellus* together with Manse-AKH [26], and in the current study from the CC extract of the crambid moth *C. culmella* (Figure 8). The accessible genome of *C. culmella* was mined for AKHs here and had predicted the synthesis of Manse-AKH and Chipa-AKH, respectively, from two AKH precursors. The same peptide pair was predicted from precursors in *Chilo suppressalis* but not chemically confirmed yet [29]. Our current MS investigations confirmed that these AKHs are produced in *C. culmella* and further revealed the presence of Vanca-AKH—the unusually processed non-amidated, elongated form arising from the Manse-AKH preprohormone (Table 1). A genome assembly from another crambid moth, the rice leaffolder *Cnaphalocris medinalis*, is available in NCBI and was also mined for AKH precursors in the current study, despite not having any biological material at our disposal for definitive investigations. Only one AKH precursor (sequence ID CM026294.1, NCBI database) that encodes for a mature Manse-AKH could be located. The Manse-AKH preprohormone precursor sequence is very similar among the five Crambidae moth species (Table 2).

To date, chemical and in silico investigations into the AKH complement of crambid moths span the six species discussed above. Thus far, *C. medinalis* is the only one of these species with a single AKH precursor (for the nonapeptide Manse-AKH), whereas the other crambid moths produce/potentially synthesize Manse-AKH and the decapeptide Chipa-AKH (in three species) or Ostnu-AKH (in two species) ([26,29], the current study). It is envisaged that gene duplication has taken place and a further evolutionary trend was the modification from glutamine to asparagine. Whether a second AKH precursor is, indeed, not present in *C. medinalis* or whether it could not be mined due to errors in the assembled genome is currently not clear.

The predicted sequence for an AKH with conventionally blocked termini in the termite *K. flavicollis* (Figure 1A) was confirmed in the current study via MS analyses with corpora cardiaca extracts from the tiny termite species. The identified peptide, code-named Manto-CC, was first found in a species of the newest insect order Mantophasmatodea [30], and has since been detected in other members of this order [31]. Manto-CC was also predicted but never shown by analytical methods, to occur in three species of moss bugs (order Hemiptera, suborder Coleorrhyncha) [32]. In termites, this peptide has only been detected, to date, in this one species of Kalotermitidae, whereas other family members synthesize the AKH code-named Peram-CAH-I [10], which differs from Manto-CC at position 7 (glycine to asparagine exchange). A possible molecular and phylogenetic relationship of AKHs in the order Blattodea, of which the termites are part, is given in a manuscript in preparation [33] based on AKH and AKH receptor sequences of termites and cockroaches. For more definite phylogenies, the AKH gene of *K. flavicollis* was cloned, and the results support our assignment of Manto-CC as the AKH for this species [33].

An AKH that was predicted from the precursor of the robber fly *D. diadema* [25] could not be shown to occur in *P. tapulus*, which we investigated in the current study; instead, a structurally very similar and related peptide with a Ser5, and not the Thr5 predicted in *D. diadema*, was identified in *P. tapulus* (compare Figure 1B and Figure 4). It is a novel octapeptide, which we have code-named Pegta-AKH. In addition, the CC of the robber fly under study synthesizes the octapeptide code-named Volpe-CC (Figure 3), formerly found in the dipteran family Syrphidae [25]. Volpe-CC differs structurally only by a Tyr7 residue instead of the Val7 in Pegta-AKH (Table 1). Thus, gene duplication has almost certainly taken place here and a point mutation.

The predicted AKH of *D. diadema* is also predicted from genomic data to be present in the cat flea *Ctenocephalides felis* (NCBI database XP_026477356.1), an insect that belongs to the order Siphonaptera. According to the newest phylogenomic research on Diptera [34], the orders Siphonaptera and Mecoptera are the closest relatives to the order Diptera. Hence, it might not be so surprising that the dipteran *D. diadema* and the siphonapteran *C. felis* share the same AKH.

**Figure 9:**
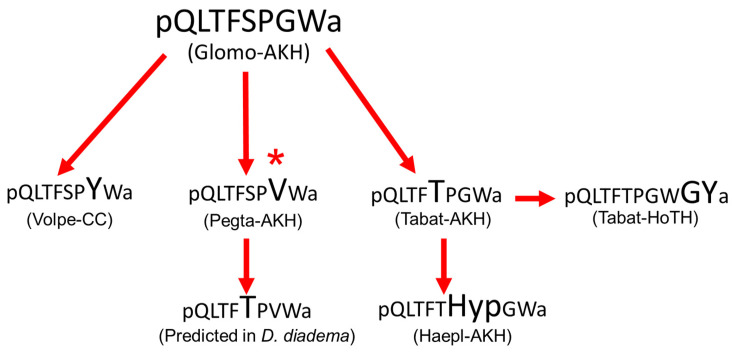
Hypothetical molecular evolution of adipokinetic peptides in Diptera. Glomo-AKH is assumed as ancestral peptide for this order. The amino acid substitution in each peptide is indicated in a larger font than the peptide from which it is hypothetically derived. All substitutions are point mutations except the change from Glomo-AKH to the novel Pegta-AKH. * The switch from Gly7 to Val7 requires two base changes.

We also analyzed the AKHs of the tabanid *H. pluvialis* (Table 1) and could confirm the two AKHs previously found in *T. atratus*: the octapeptide Tabat-AKH and the decapeptide Tabat-HoTH, which is an extended form of Tabat-AKH (Figure 5). Tabat-AKH differs from the novel Pegta-AKH only by a glycine to valine modification at position 7. The infraorder Tabanomorpha is closely related to the superfamily Asiloidea [34] and this is reflected in some structural relatedness of the AKHs of the two taxa. Interestingly, there is yet another AKH in the horse fly *H. pluvialis*: this is a posttranslationally produced octapeptide, where the proline residue at position 6 of Tabat-AKH is hydroxylated; we call this novel hydroxyproline containing octapeptide Haepl-AKH (Figure 5A). Presently, this is the second time hydroxyprolination has been shown for an AKH. The first example was for the green stink bug *Nezara viridula* (order Hemiptera, suborder Heteroptera) [16]. Taken together, the current study of dipteran AKHs in robber and horse flies has not only confirmed predicted peptides but has elucidated structurally novel AKHs as well. The new sequences, Pegta-AKH and Haepl-AKH, fit very well in the scheme of putative molecular evolution of dipteran AKHs as proposed recently [25]. A modified scheme depicting only the ancestral dipteran AKH, i.e., Glomo-AKH of the lower Diptera and sister order Mecoptera (see [25]), and the newly found sequences are given in Figure 9. Clearly, the close relatedness of the robber fly AKHs (Volpe-CC, Pegta-AKH, *D. diadema* predicted AKH) is obvious, as well as those of the horse flies (Tabat-AKH, Tabat-HoTH, Haepl-AKH).

With more and more unannotated genomic and transcriptomic data available, it becomes important to first screen the databases for potential AKHs and then test the biological material for the putative peptides for further downstream applications, such as phylogenetic analyses, physiological investigations, and biochemical studies. This database screening step assists the analytical chemist in the targeted search for the identified AKH structures, and in this way saves material and speeds up the discovery process considerably.

## 4. Materials and Methods

### 4.1. Insects and CC Preparation

Corpora cardiaca (CC) were dissected from adult insects of indeterminate age. Specimens of the termite *Kalotermes flavicollis* (Blattodea, Isoptera, Kalotermitidae) were a gift from Prof. D.P. McMahon (Federal Institute of Materials Research and Testing, BAM, Berlin, Germany). Specimens of the horse fly *Haematopota pluvialis* (Diptera, Brachycera, Tabanomorpha, Tabanidae) were collected by netting at a farm close to Orlamünde (Thuringia, Germany). Adults of the robber fly *Pegesimallus tapulus* (Diptera, Brachycera, Asiloidea, Asilidae) were collected by netting in a private garden in Cape Town (Western Cape Province, South Africa). Pupae of the European maize borer *Ostrinia nubilalis* were a gift of Prof. F. Marec (Biology Centre, Czech Academy of Sciences, Institute of Entomology, České Budějovice, Czechia); newly-eclosed adults were used for gland dissection. Adult specimens of the garden grass-veneer *Chrysoteuchia culmella* were collected by netting in grasslands close to Orlamünde (Thuringia, Germany); both *O. nubilalis* and *C. culmella* are moth species: Lepidoptera, Obtectomera, Papilionoidea, Crambidae.

CC from individual insects (*n* = 8, 11, 13, 17, and 25, respectively, for *K. flavicollis*, *H. pluvialis*, *C. culmella*, *P. tapulus*, and *O. nubilalis*), were dissected with the aid of a stereomicroscope at 20- to 40-fold magnification. The glands from the same species were pooled in a microcentrifuge tube containing 80% methanol, extracted by approved methods [35], and dried in a vacuum centrifuge.

### 4.2. Mining of AKH Sequences from Publicly Available Databases

The primary sequence of AKH family peptides in blattodean, dipteran, and lepidopteran species was investigated by MS (see Section 4.3). To facilitate the identification and analyses of AKHs from crude glandular extracts via target-MS, we identified putative AKH sequences from related dipteran species via literature searches (i.e., from previously published texts), as well as via bioinformatics. In the case of the lepidopteran species under investigation, we performed bioinformatic searches in the available genomes. Such in silico searches of protein, genomic, and/or EST databases were conducted to identify translated amino acid sequences and transcripts encoding putative AKH peptide precursors.

The putative AKH sequences were obtained via homology searches using BLAST from the National Center for Biotechnology Information site (https://blast.ncbi.nlm.nih.gov/, accessed on between 6 April 2022 and 13 June 2022); AKH peptide precursors from related species were used as BLAST query. For all searches resulting in sequence identifications, the BLAST score and BLAST-generated E-value for significant alignment were considered. From the search results, the AKH peptide sequence contained within the deduced prepro-hormones were predicted from homology to known insect AKH analogs.

### 4.3. Synthetic Peptides

The novel peptides from this study code-named Ostnu-AKH, Haepl-AKH, and Pegta-AKH, as well as Manse-AKH, Chipa-AKH, and Volpe-CC were synthesized by Pepmic Co., Ltd. (Suzhou, China). The two other peptides, Tabat-AKH and Tabat-HoTH, had been custom-synthesized earlier by Peninsula Laboratories (Belmont, CA, USA). They were prepared at ~1 pmol/µL in 50/50 *v*/*v* methanol and 0.1% formic acid containing 5% acetonitrile.

### 4.4. Structure Elucidation by LC-MS

The dried extracts were dissolved in 10 µL methanol followed by 10 µL 0.1% formic acid containing 5% acetonitrile. For LC-MS/MS, Synapt G2 Si (Q-TOF with ion mobility) coupled to M-Class nanoUPLC (Waters Corp., Manchester, UK) was employed using C18 µPAC columns (trapping and 50 cm analytical; PharmaFluidics, Ghent, Belgium) with a 30 min gradient (10–60%; solvent system 100% water versus 100% acetonitrile, both containing 0.1% formic acid; 0.5–1 µL injection volume). AKH candidates were identified by target-MS (MS/MS on pre-selected *m*/*z* values) for eligible known peptide masses from related insect species according to references [25,26] and putative sequence information gleaned from bioinformatic searches (see Section 4.2) using their singly- and doubly-charged ions, as well as by screening with low/high collision energy switching for the gas phase loss of the tryptophan immonium ion in data-independent runs. Moreover, AKH candidates were obtained by manual interrogation of data-dependent runs and the use of marker fragment ions discovered for proline-containing AKHs (manuscript in preparation) [36]. Sequence ion assignment was used as calculated by the MassLynx spectrometer software, which treats pyroglutamate (Pyr) as terminal modification rather than a modified amino acid thus creating a label shift for ion assignment by one in comparison to the amino acid number. The fragment ion tables for the spectra shown here are available in the Supplementary Materials for clarification. Peptide sequences were validated by comparison to the performance of the respective synthetic peptides.

## Figures and Tables

**Table 1:** AKHs determined in this study using high-resolution MS. MW—molecular weight, RT—retention time.

Insect Species	Primary Sequence	MW	RT (min)	Peptide Name
*Kalotermes flavicollis*	pQ VNFSPG W amide	915.424	32.1	Manto-CC
*Pegesimallus tapulus*	pQ LTFSPY W amide	1022.486	34.6	Volpe-CC
*Pegesimallus tapulus*	pQ LTFSPV W amide	958.491	36.8	Pegta-AKH
*Haematopota pluvialis*	pQ LTFTPG WGY amide	1150.545	35.4	Tabat-HoTH
*Haematopota pluvialis*	pQ LTFTPG W amide	930.460	34.9	Tabat-AKH
*Haematopota pluvialis*	pQ LTFTHypG W amide	946.455	33.4	Haepl-AKH
*Ostrinia nubilalis*	pQ LTFTSS WG amide	1007.471	34.3	Manse-AKH
*Ostrinia nubilalis*	pQ LTFTSS WGGK	1193.572	32.6	Vanca-AKH
*Ostrinia nubilalis*	pQ LTFSTG WGQ amide	1105.519	32.8	Ostnu-AKH
*Chrysoteuchia culmella*	pQ LTFTSS WG amide	1007.471	33.8	Manse-AKH
*Chrysoteuchia culmella*	pQ LTFTSS WGGK	1193.572	32.1	Vanca-AKH
*Chrysoteuchia culmella*	pQ LTFSTG WGN amide	1091.504	35.0	Chipa-AKH

**Table 2:** Comparison of Manse-AKH precursors in Crambidae moths.

Species	Predicted AKH Precursor
*C. medinalis*	MYKYLVILVLVACICVATEA**QLTFTSSWG**GKRSGAITGGVSCRNDEAIAAIYKMIQ
*C. culmella*	MHKFLALLVIVACVCMAVDA**QLTFTSSWG**GKRAAIAGTVSCRNDDAIAAIYKMIQ
*O. nubilalis*	MYKFLAVFVLIACMCLVAEA**QLTFTSSWG**GKRAAIAGTVSCRNDEAIAAIYKMIQVRTPLF
*O. furnacalis*	MYKFLAVFVLIACMCLVAEA**QLTFTSSWG**GKRAAIAGTVSCRNDEAIAAIYKMIQNEAERFILCQKP
*C. suppressalis*	MYKLLVLLVVVACVCAAVEA**QLTFTSSWG**GKRAAIAGTVSCRNDEAIAAIYKMIQNEAERFILCQKP

Species and Accession/Assembly No.: *Cnaphlocrocis medinalis* (CM026294.1), *Chrysoteuchia culmella* (OU342662.1), *Ostrinia nubilalis* (SWFO01000347.1), *Ostrinia furnacalis* (XP_028164252.1), *Chilo suppressalis* (ALM30296.1).

## Data Availability

All data are given in the results and supplementary sections.

## Supplementary Materials

The following supporting information can be downloaded at: https://www.mdpi.com/article/10.3390/molecules27196469/s1, Supplement: raw and validation spectra. Figure S1. MS/MS spectra of the singly- and the doubly-charged ions of a peptide assigned to Manto-CC in *K. flavicollis* and the calculation of the expected fragment ions for sequence pQVNFSPGWa using MassLynx (blocked termini: pQ and amidation). Figure S2. MS/MS spectra of the doubly-charged ions of the peptide assigned to Manto-CC in *K. flavicollis*. (A) Standard peptide and (B) CC extract were run with the same MS/MS method. Figure S3. MS/MS spectrum of the singly-charged ion of the peptide assigned to Volpe-CC in *P. tapulus* and the calculation of the expected fragment ions for sequence pQLTFSPYWa using MassLynx (blocked termini: pQ and amidation). Figure S4. MS/MS spectra of the singly-charged ion of the peptide assigned to Volpe-CC in *P. tapulus*. A) CC extract and B) standard peptide were run with slightly different collision energy (17–26 eV and 17–25 eV), but the fragments are identical. Figure S5. MS/MS spectra of the singly- and doubly-charged ions of the peptide detected at *m*/*z* 959.52 tentatively assigned to sequence pQLTFSPVWa in *P. tapulus* and the calculation of the expected fragment ions using MassLynx (blocked termini: pQ and amidation). Figure S6. MS/MS spectra of the singly-charged ions of the peptide detected at *m*/*z* 959.52 assigned to sequence pQLTFSPVWa in *P. tapulus*, code-named Pegta-AKH. (A) Standard peptide and (B) CC extract were analyzed with the same MS/MS method and instrumentation. Figure S7. MS/MS spectra of the singly- and doubly-charged ions of the peptide detected at *m*/*z* 1151.55 assigned to sequence pQLTFTPGWGYa for Tabat-HoTH in *H. pluvialis* and the calculation of the expected fragment ions using MassLynx (blocked termini: pQ and amidation). Figure S8. MS/MS spectra of the singly-charged ions of the peptide detected at *m*/*z* 1151.55 assigned to sequence pQLTFTPGWGYa for Tabat-HoTH in *H. pluvialis*. (A) CC extract and (B) standard peptide were run with the same collision energy ramp. Figure S9. MS/MS spectra of the doubly-charged ions of the related peptides detected in *H. pluvialis* at *m*/*z* 931.47 and 947.46 assigned, respectively, to Tabat-AKH (pQLTFTPGWa) and its hydroxyproline derivative. The expected fragment ions for each of these peptide sequences were calculated using MassLynx (blocked termini: pQ and amidation). Figure S10. MS/MS spectra of the singly-charged ions of the peptide detected at *m*/*z* 931.47 assigned to sequence pQLTFTPGWa for Tabat-AKH in *H. pluvialis*. (A) Standard peptide and (B) CC extract were analyzed with the same MS/MS method. Figure S11. MS/MS spectra of the singly-charged ions of the peptide detected at 947.46 assigned to the hydroxyproline derivative of Tabat-AKH (Haepl-AKH) in *H. pluvialis*. (A) Standard peptide (33.3 min) and (B) CC extract (33.3 min) were analyzed with the same MS/MS method. Figure S12. MS/MS spectra of the singly- and the doubly-charged ions of the peptide detected at *m*/*z* 1194.58 assigned to sequence pQLTFTSSWGGK for Vanca-AKH in *O. nubilalis* and the calculation of the expected fragment ions using MassLynx (blocked terminus: pQ). Figure S13. MS/MS spectra of the singly- and the doubly-charged ions of the peptide detected at m/z 1008.48 assigned to sequence pQLTFTSSWGa for Manse-AKH in *O. nubilalis* and the calculation of the expected fragment ions using MassLynx (blocked termini: pQ and amidation). Figure S14. MS/MS spectra of the singly-charged ions of the peptide detected at *m*/*z* 1008.48 assigned to sequence pQLTFTSSWGa for Manse-AKH in *O. nubilalis*. (A) Standard peptide (34.7 min) and (B) CC extract (34.8 min) were with slightly different collision energy (15–20 eV and 17–26 eV), but the fragments are identical. Figure S15. MS/MS spectra of the singly- and the doubly-charged ions of the peptide detected at *m*/*z* 1106.53 assigned to sequence pQLTFSTGWGQa for Ostnu-AKH in *O. nubilalis* and the calculation of the expected fragment ions using MassLynx (blocked termini: pQ and amidation). Figure S16. MS/MS spectra of the singly- and the doubly-charged ions of the peptide detected at *m*/*z* 1106.53 assigned to sequence pQLTFSTGWGQa for Ostnu-AKH in *O. nubilalis*. (A) Standard peptide (34.2 min) and (B) CC extract (34.3) were run with the same MS/MS method. Figure S17. MS/MS spectra of the singly- and the doubly-charged ions of the peptides detected for Manse- and Vanca-AKH in *C. culmella*. Figure S18. MS/MS spectra of the singly- and the doubly-charged ions of the peptides detected for Manse-AKH in *C. culmella*. (A) Standard peptide and (B) CC extract were run with slightly different collision energy (15–20 eV and 17–26 eV), but the fragments are identical. Figure S19. MS/MS spectra of the singly- and the doubly-charged ions of the peptide detected at *m*/*z* 1092.57 assigned to sequence pQLTFSTGWGNa for Chipa-AKH in *C. culmella* and the calculation of the expected fragment ions using MassLynx (blocked termini: pQ and amidation). Figure S20. MS/MS spectra of the singly-charged ions of the peptide detected at *m*/*z* 1092.57 assigned to sequence pQLTFSTGWGNa for Chipa-AKH in *C. culmella*. (A) CC extract and (B) standard peptide were analyzed with the same MS/MS method. Figure S21. LC-chromatograms for the base peak in the overview scans for the CC extract of (A) *P. tapulus*, (B) *C. culmella* (C) *O. nubilalis*, (D) *K. flavicollis*.

## Sample Availability

Samples were only available to the authors.
