# Supplementary material for: Mass Spectrometric Proof of Predicted Peptides: Novel Adipokinetic Hormones in Insects
Source: Molecules. 2022 Oct 1;27(19):6469. doi: 10.3390/molecules27196469 (PMC9573411; doi:10.3390/molecules27196469)
Supplement: Supplementary file 1 [file molecules-27-06469-s001.zip › molecules-1889640-supplementary.pdf]

molecules

MDPI

Article

Mass Spectrometric Proof of Predicted Peptides: Novel Adipokinetic Hormones in Insects

Heather G. Marco [1,†], Simone König [2,†] and Gerd Gäde [1,*]

[1] Department of Biological Sciences, University of Cape Town, Private Bag, Rondebosch, Cape Town ZA-7700, South Africa; heather.marco@uct.ac.za
[2] IZKF Core Unit Proteomics, Interdisciplinary Center for Clinical Research, University of Münster, Röntgenstr. 21, 48149 Münster, Germany; koenigs@uni-muenster.de
* Correspondence: gerd.gade@uct.ac.za; Tel.: +27-21-650-3615
† These authors contributed equally to this paper.

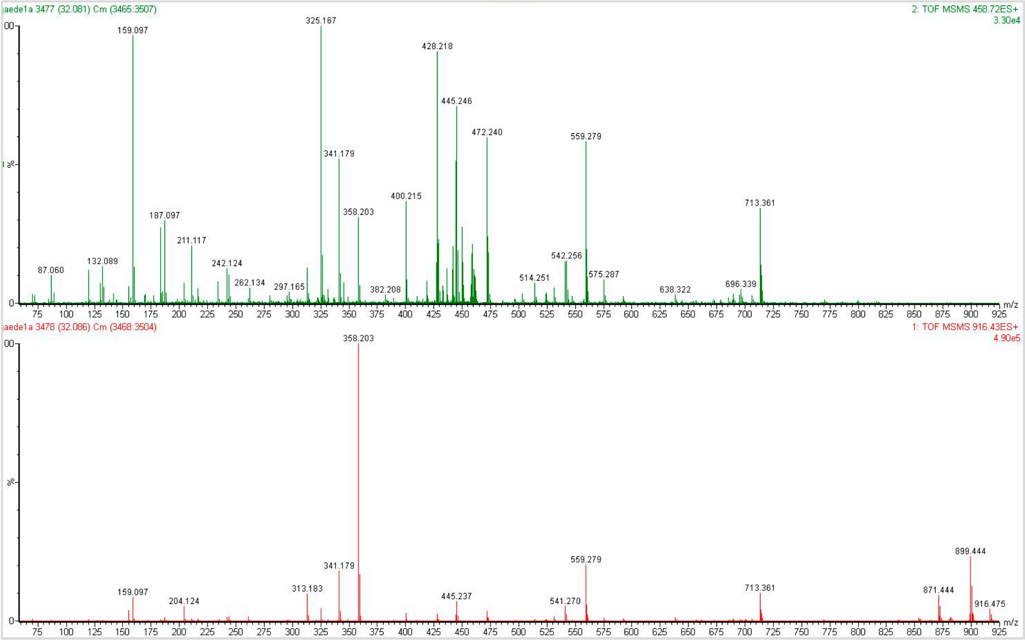

b	211.108	325.151	472.220	559.252	656.304	713.326	-
i	72.081	87.056	120.081	60.045	70.066	30.034	159.092
b~	193.098	307.141	454.209	541.241	638.294	695.315	-
b*	194.082	308.125	455.193	542.225	639.278	696.299	-
	1	2	3	4	5	6	7
	Val	Asn	Phe	Ser	Pro	Gly	Trp
	7	6	5	4	3	2	1
y"	-	706.331	592.288	445.220	358.188	261.135	204.114
y~	-	688.321	574.278	427.209	340.177	243.125	186.103
y*	-	689.305	575.262	428.193	341.161	244.109	187.087

Figure S1. MS/MS spectra of the singly- and the doubly-charged ions of a peptide assigned to Manto-CC in *K. flavicollis* and the calculation of the expected fragment ions for sequence pQVNF-SPGWa using MassLynx (blocked termini: pQ and amidation).

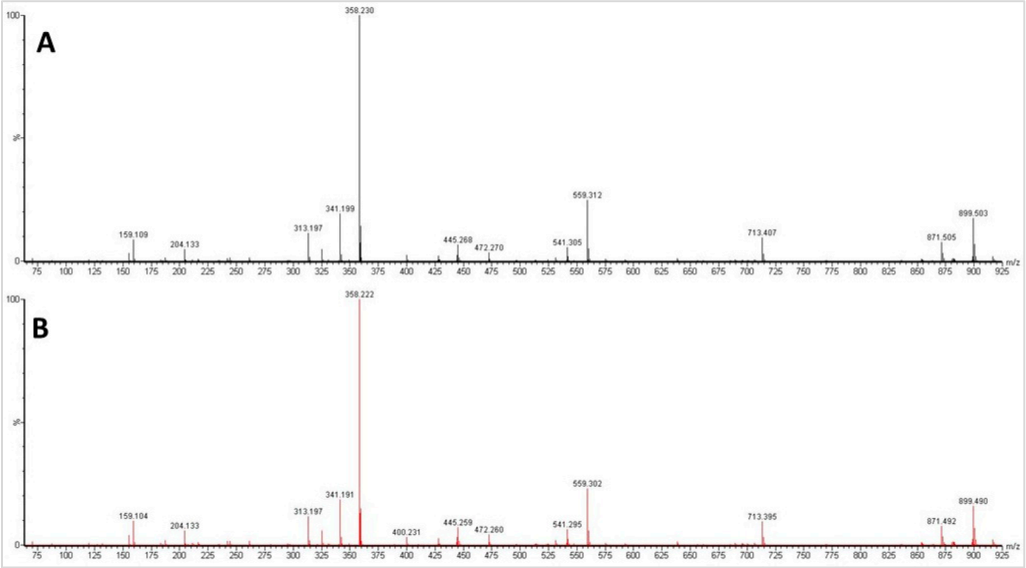

Figure S2. MS/MS spectra of the doubly-charged ions of the peptide assigned to Manto-CC in *K. flavicollis*. A) Standard peptide and B) CC extract were run with the same MS/MS method.

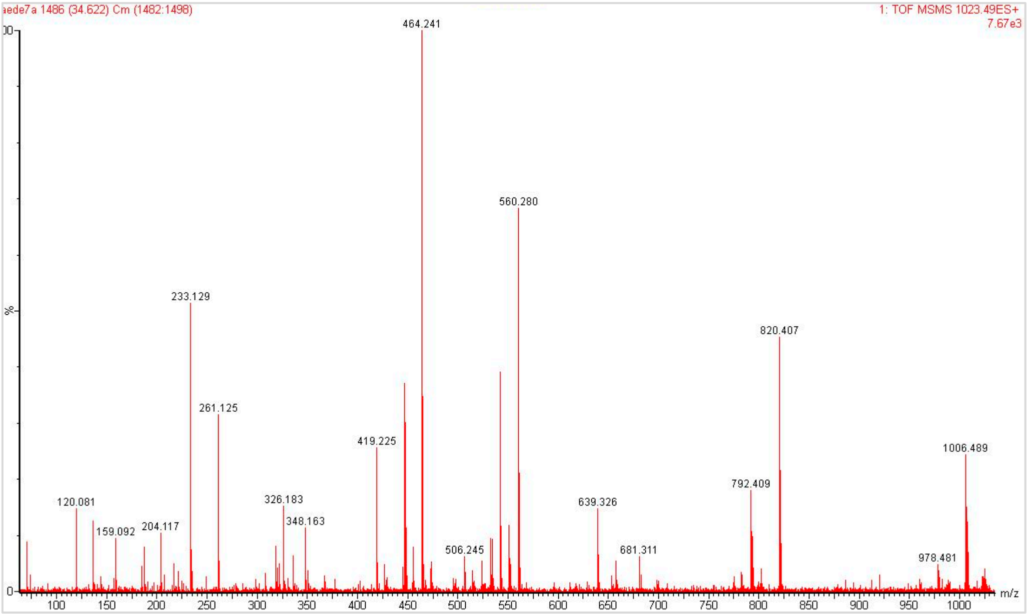

b	225.124	326.172	473.240	560.272	657.325	820.388	-
i	86.097	74.061	120.081	60.045	70.066	136.076	159.092
b~	207.113	308.161	455.229	542.262	639.314	802.378	-
b*	208.097	309.145	456.214	543.246	640.298	803.362	-
	1	2	3	4	5	6	7
	Leu	Thr	Phe	Ser	Pro	Tyr	Trp
	7	6	5	4	3	2	1
y"	-	799.378	698.330	551.262	464.230	367.177	204.114
y~	-	781.367	680.320	533.251	446.219	349.166	186.103
y*	-	782.351	681.304	534.235	447.203	350.150	187.087

Figure S3. MS/MS spectrum of the singly-charged ion of the peptide assigned to Volpe-CC in *P. tapulus* and the calculation of the expected fragment ions for sequence pQLTFSPYWa using MassLynx (blocked termini: pQ and amidation).

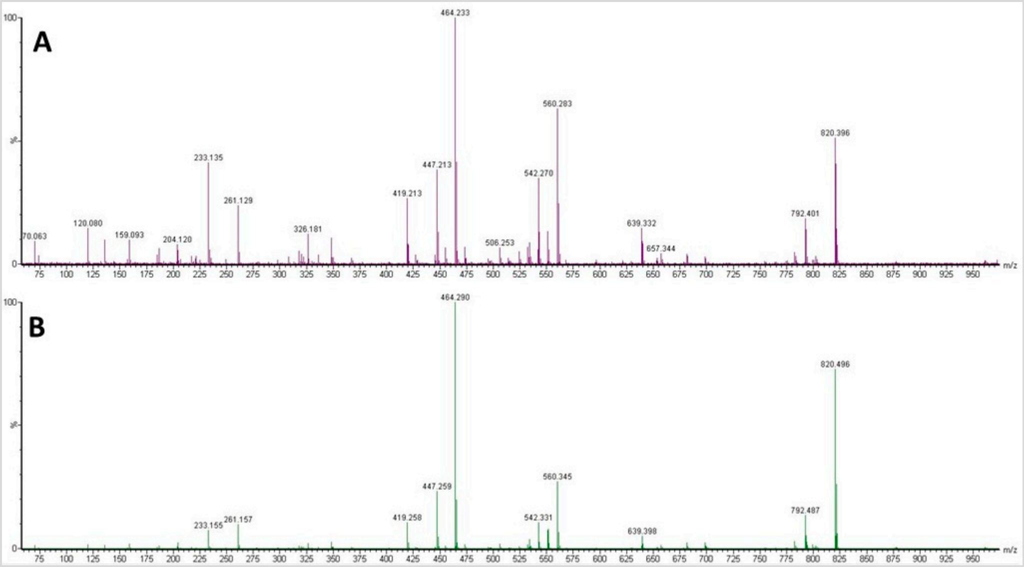

Figure S4. MS/MS spectra of the singly-charged ion of the peptide assigned to Volpe-CC in *P. tapulus*. A) CC extract and B) standard peptide were run with slightly different collision energy (17-26 eV and 17-25 eV), but the fragments are identical.

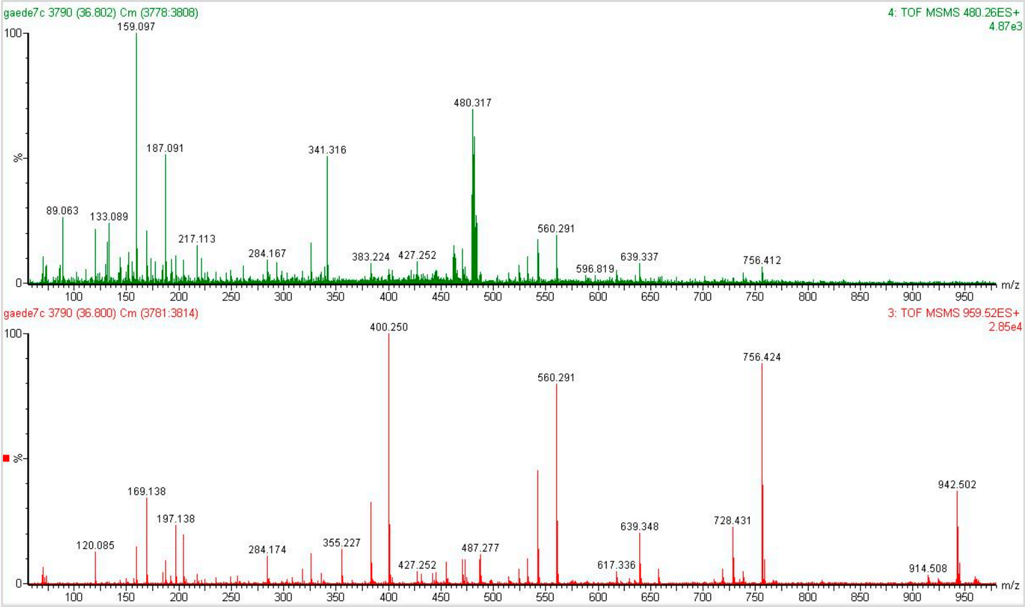

b	225.124	326.172	473.240	560.272	657.325	756.393	-
i	86.097	74.061	120.081	60.045	70.066	72.081	159.092
b~	207.113	308.161	455.229	542.262	639.314	738.383	-
b*	208.097	309.145	456.214	543.246	640.298	739.367	-
	1	2	3	4	5	6	7
	Leu	Thr	Phe	Ser	Pro	Val	Trp
	7	6	5	4	3	2	1
y"	-	735.383	634.335	487.267	400.235	303.182	204.114
y~	-	717.372	616.325	469.256	382.224	285.172	186.103
y*	-	718.356	617.309	470.240	383.208	286.156	187.087

Figure S5. MS/MS spectra of the singly- and doubly-charged ions of the peptide detected at *m/z* 959.52 tentatively assigned to sequence pQLTFSPVWa in *P. tapulus* and the calculation of the expected fragment ions using MassLynx (blocked termini: pQ and amidation).

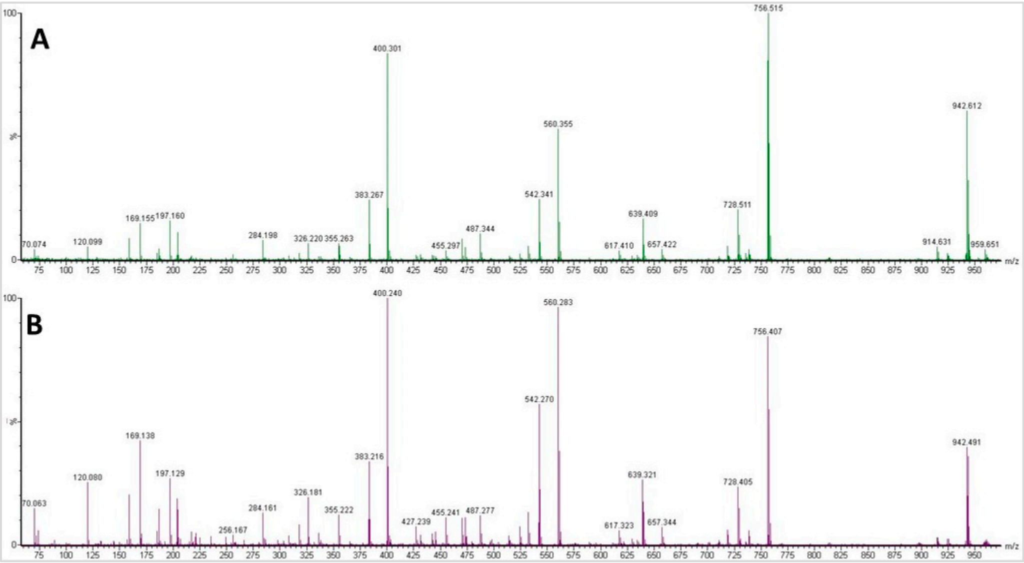

Figure S6. MS/MS spectra of the singly-charged ions of the peptide detected at *m/z* 959.52 assigned to sequence pQLTFSPVWa in *P. tapulus*, code-named Pegta-AKH. A) Standard peptide and B) CC extract were analyzed with the same MS/MS method and instrumentation.

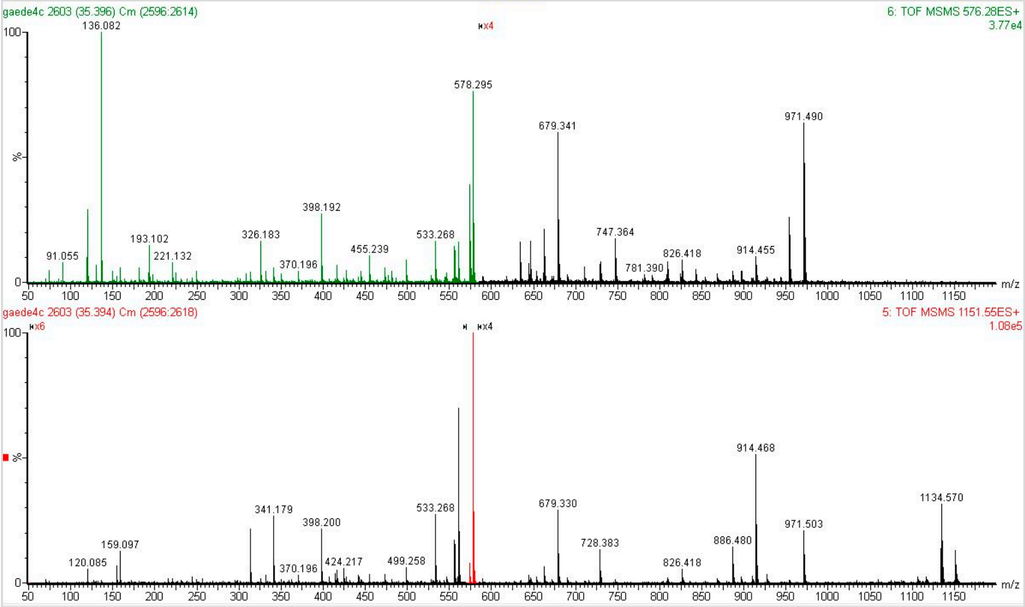

b	225.124	326.172	473.240	574.288	671.341	728.362	914.441	971.463	-
i	86.097	74.061	120.081	74.061	70.066	30.034	159.092	30.034	136.076
b~	207.113	308.161	455.229	556.277	653.330	710.351	896.431	953.452	-
b*	208.097	309.145	456.214	557.261	654.314	711.335	897.415	954.436	-
	1	2	3	4	5	6	7	8	9
	Leu	Thr	Phe	Thr	Pro	Gly	Trp	Gly	Tyr
	9	8	7	6	5	4	3	2	1
y"	-	927.436	826.389	679.320	578.273	481.220	424.198	238.119	181.098
y~	-	909.426	808.378	661.310	560.262	463.209	406.188	220.109	163.087
y*	-	910.410	809.362	662.294	561.246	464.193	407.172	221.093	164.071

Figure S7. MS/MS spectra of the singly- and doubly-charged ions of the peptide detected at *m*/*z* 1151.55 assigned to sequence pQLTFTPGWGYa for Tabat-HoTH in *H. pluvialis* and the calculation of the expected fragment ions using MassLynx (blocked termini: pQ and amidation).

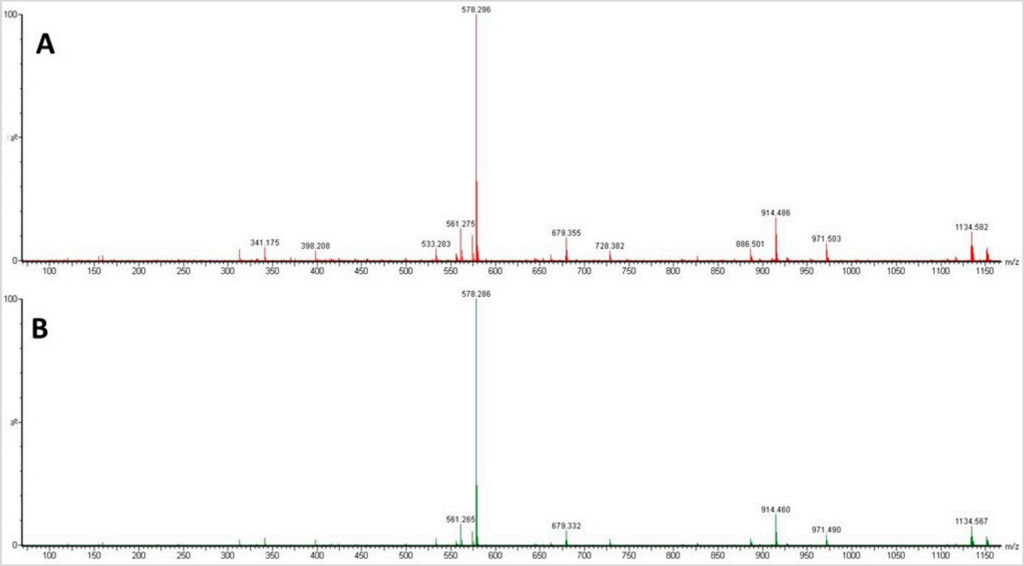

Figure S8. MS/MS spectra of the singly-charged ions of the peptide detected at *m*/*z* 1151.55 assigned to sequence pQLTFTPGWGYa for Tabat-HoTH in *H. pluvialis.* A) CC extract and B) standard peptide were run with the same collision energy ramp.

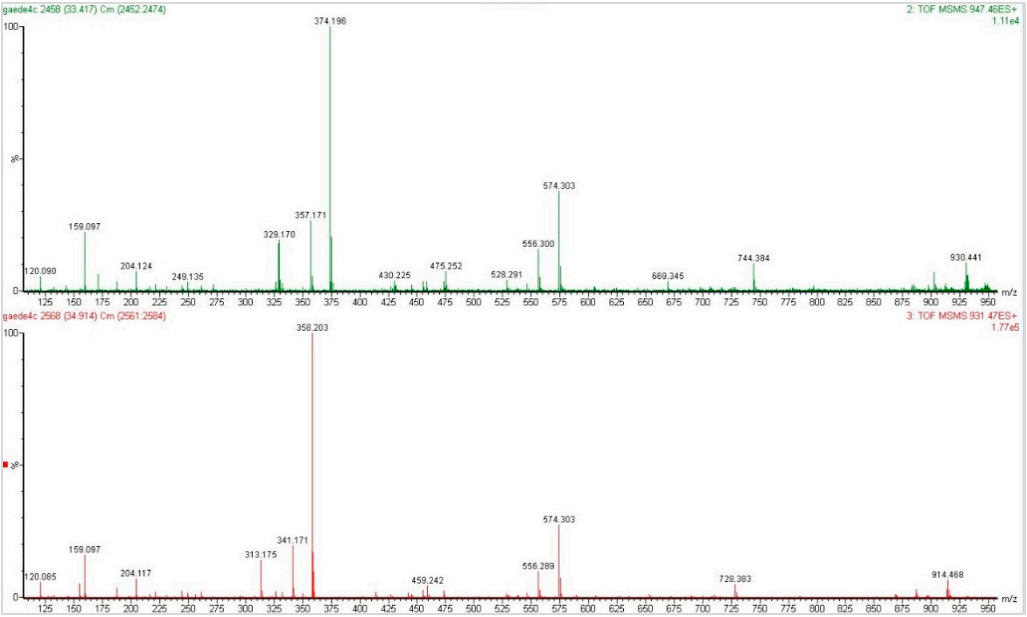

b	225.124	326.172	473.240	574.288	687.335	744.357	-
i	86.097	74.061	120.081	74.061	86.061	30.034	159.092
b~	207.113	308.161	455.229	556.277	669.325	726.346	-
b*	208.097	309.145	456.214	557.261	670.309	727.330	-
	1	2	3	4	5	6	7
	**Leu**	**Thr**	**Phe**	**Thr**	**Hyp**	**Gly**	**Trp**
	7	6	5	4	3	2	1
y"	-	723.347	622.299	475.230	374.183	261.135	204.114
y~	-	705.336	604.288	457.220	356.172	243.125	186.103
y*	-	706.320	605.272	458.204	357.156	244.109	187.087

b	225.124	326.172	473.240	574.288	671.341	728.362	-
i	86.097	74.061	120.081	74.061	70.066	30.034	159.092
b~	207.113	308.161	455.229	556.277	653.330	710.351	-
b*	208.097	309.145	456.214	557.261	654.314	711.335	-
	1	2	3	4	5	6	7
	**Leu**	**Thr**	**Phe**	**Thr**	**Pro**	**Gly**	**Trp**
	7	6	5	4	3	2	1
y"	-	707.352	606.304	459.236	358.188	261.135	204.114
y~	-	689.341	588.293	441.225	340.177	243.125	186.103
y*	-	690.325	589.277	442.209	341.161	244.109	187.087

Figure S9. MS/MS spectra of the doubly-charged ions of the related peptides detected in *H. pluvialis* at *m*/*z* 931.47 and 947.46 assigned, respectively, to Tabat-AKH (pQLTFTPGWa) and its hydroxyproline derivative. The expected fragment ions for each of these peptide sequences were calculated using MassLynx (blocked termini: pQ and amidation).

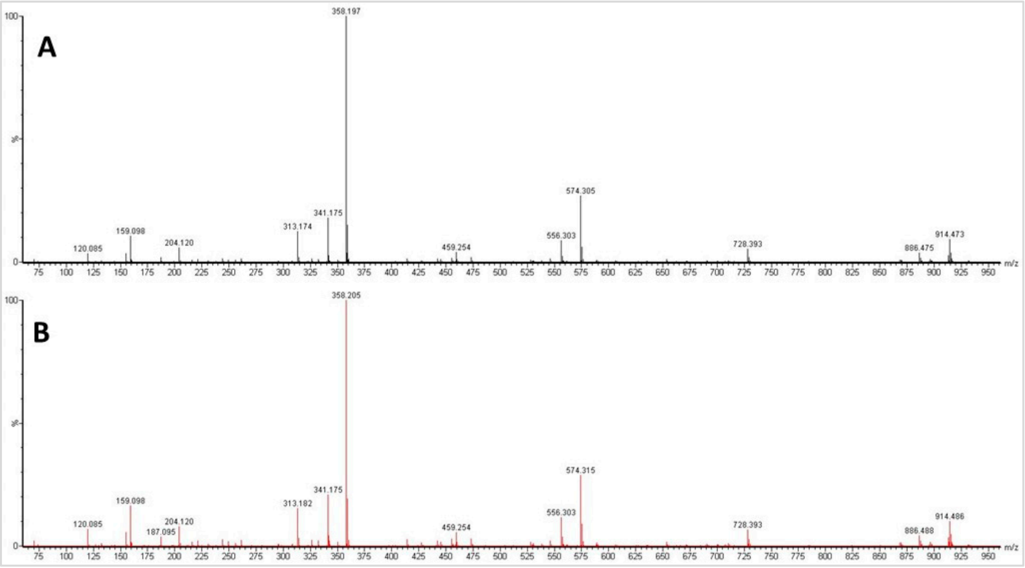

Figure S10. MS/MS spectra of the singly-charged ions of the peptide detected at *m*/*z* 931.47 assigned to sequence pQLTFTPGWa for Tabat-AKH in *H. pluvialis.* A) Standard peptide and B) CC extract were analyzed with the same MS/MS method.

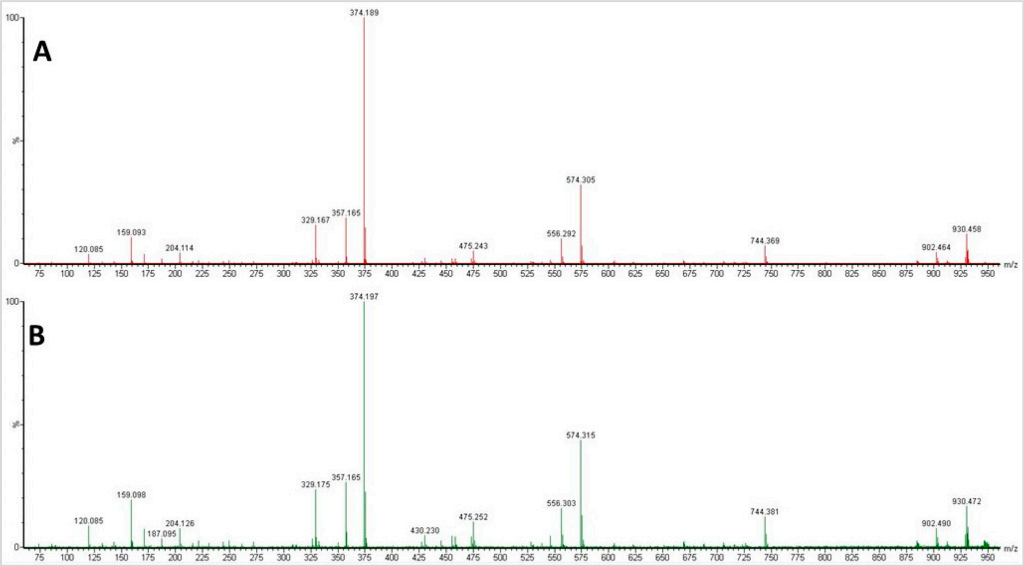

Figure S11. MS/MS spectra of the singly-charged ions of the peptide detected at 947.46 assigned to the hydroxyproline derivative of Tabat-AKH (Haepl-AKH) in *H. pluvialis.* A) Standard peptide (33.3 min) and B) CC extract (33.3 min) were analyzed with the same MS/MS method.

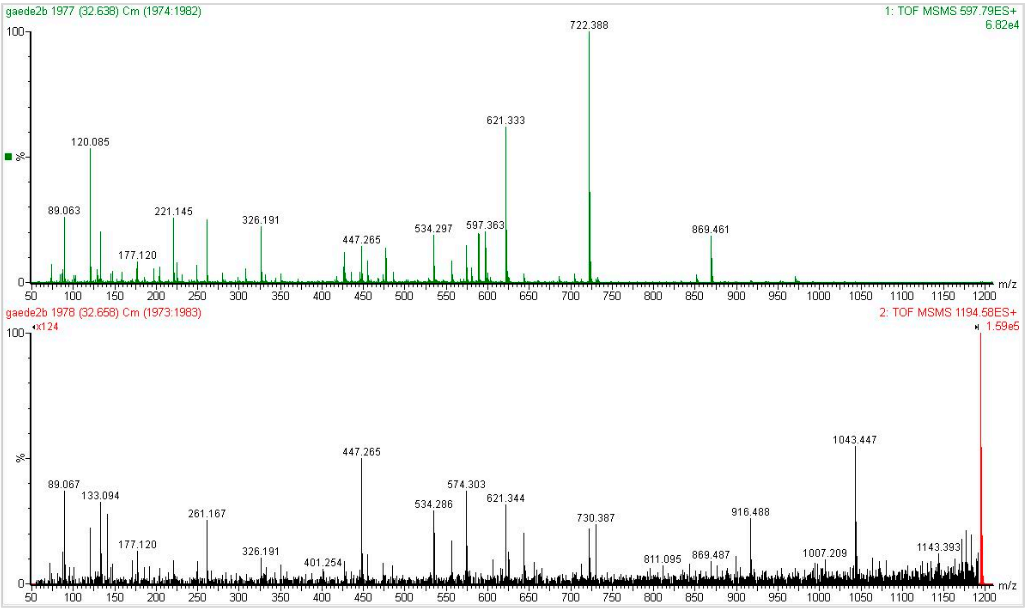

b	225.124	326.172	473.240	574.288	661.320	748.352	934.431	991.453	1048.474	-
i	86.097	74.061	120.081	74.061	60.045	60.045	159.092	30.034	30.034	101.108
b~	207.113	308.161	455.229	556.277	643.309	730.341	916.421	973.442	1030.463	-
b*	208.097	309.145	456.214	557.261	644.293	731.325	917.405	974.426	1031.448	-
	1	2	3	4	5	6	7	8	9	10
	Leu	Thr	Phe	Thr	Ser	Ser	Trp	Gly	Gly	Lys
	10	9	8	7	6	5	4	3	2	1
y"	-	970.463	869.416	722.347	621.300	534.268	447.236	261.156	204.135	147.113
y··	-	952.453	851.405	704.337	603.289	516.257	425.225	243.146	186.124	129.103
y*	-	953.437	852.389	705.321	604.273	517.241	430.209	244.130	187.108	130.087

Figure S12. MS/MS spectra of the singly- and the doubly-charged ions of the peptide detected at *m/z* 1194.58 assigned to sequence pQLTFTSSWGGK for Vanca-AKH in *O. nubilalis* and the calculation of the expected fragment ions using MassLynx (blocked terminus: pQ).

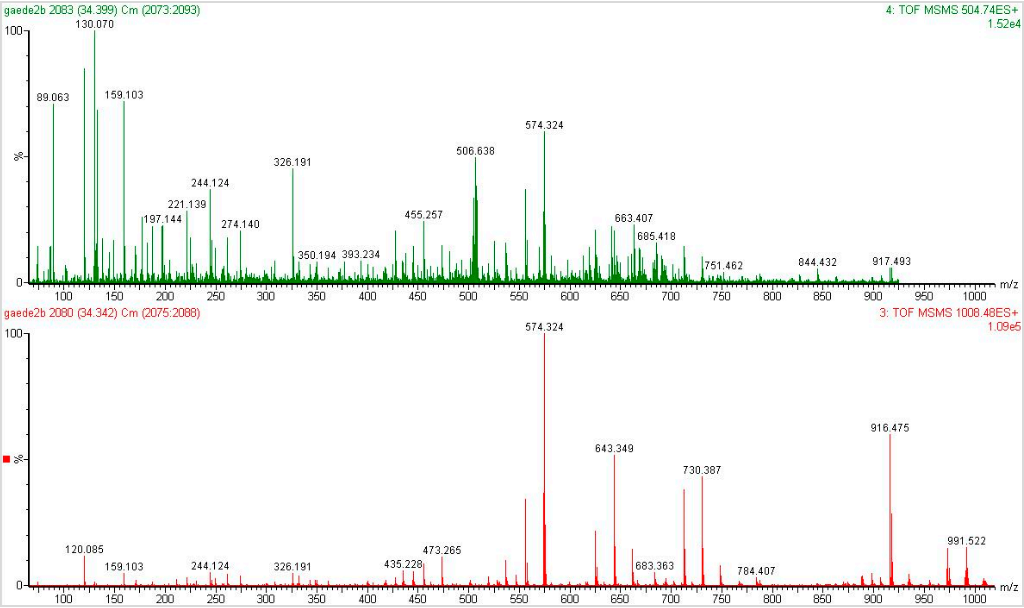

b	225.124	326.172	473.240	574.288	661.320	748.352	934.431	-
i	86.097	74.061	120.081	74.061	60.045	60.045	159.092	30.034
b~	207.113	308.161	455.229	556.277	643.309	730.341	916.421	-
b*	208.097	309.145	456.214	557.261	644.293	731.325	917.405	-
	1	2	3	4	5	6	7	8
	Leu	Thr	Phe	Thr	Ser	Ser	Trp	Gly
	8	7	6	5	4	3	2	1
y"	-	784.363	683.315	536.247	435.199	348.167	261.135	75.056
y~	-	766.352	665.305	518.236	417.189	330.157	243.125	57.045
y*	-	767.336	666.289	519.220	418.173	331.141	244.109	58.029

Figure S13. MS/MS spectra of the singly- and the doubly-charged ions of the peptide detected at *m*/*z* 1008.48 assigned to sequence pQLTFTSSWGa for Manse-AKH in *O. nubilalis* and the calculation of the expected fragment ions using MassLynx (blocked termini: pQ and amidation).

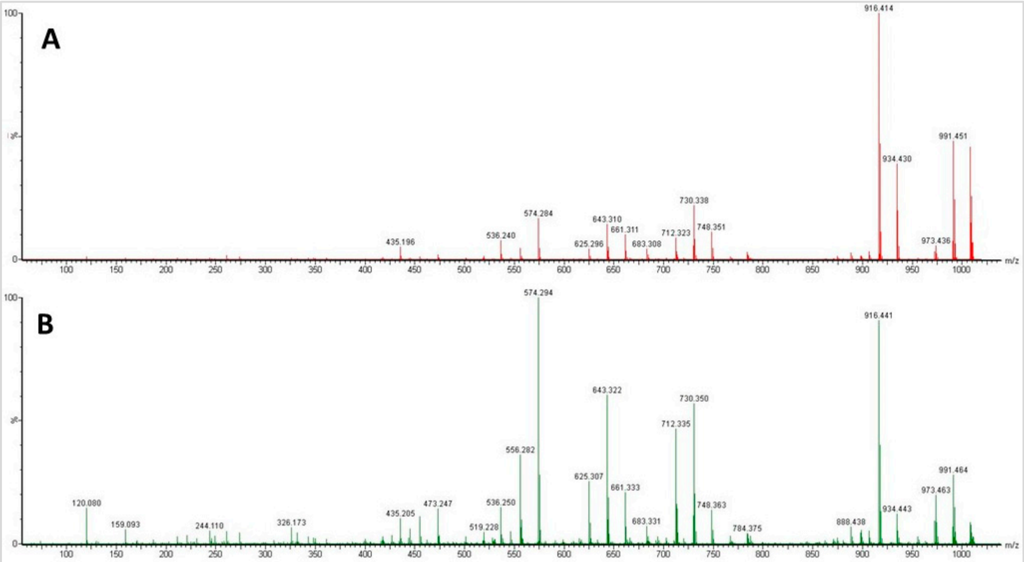

Figure S14. MS/MS spectra of the singly-charged ions of the peptide detected at *m*/*z* 1008.48 assigned to sequence pQLTFTSSWGa for Manse-AKH in *O. nubilalis.* A) Standard peptide (34.7 min) and B) CC extract (34.8 min) were with slightly different collision energy (15-20 eV and 17-26 eV), but the fragments are identical.

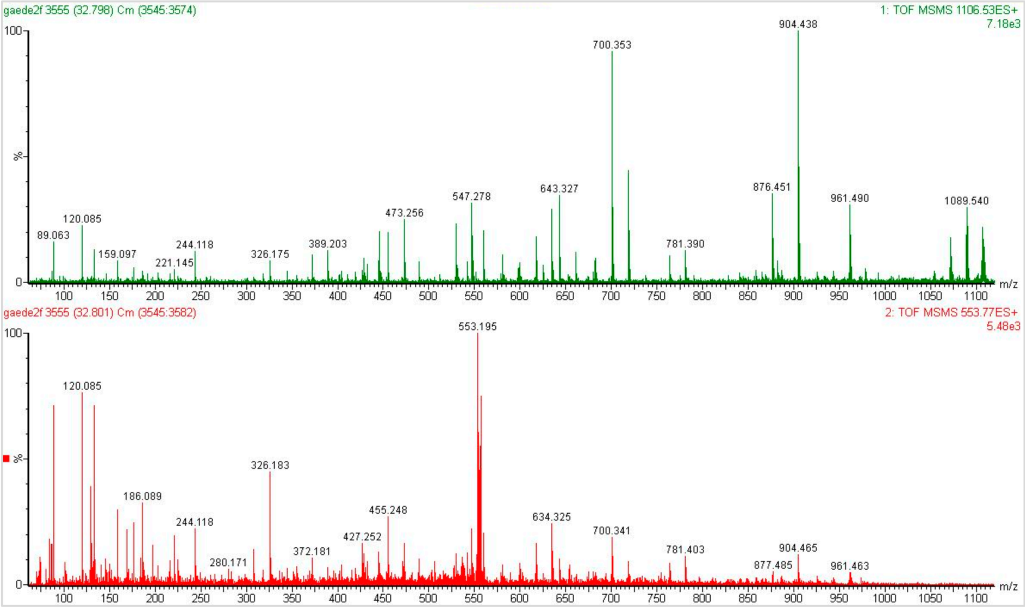

b	225.124	326.172	473.240	560.272	661.320	718.341	904.421	961.442	-
i	86.097	74.061	120.081	60.045	74.061	30.034	159.092	30.034	101.071
b~	207.113	308.161	455.229	542.262	643.309	700.331	886.410	943.431	-
b*	208.097	309.145	456.214	543.246	644.293	701.315	887.394	944.415	-
	1	2	3	4	5	6	7	8	9
	Leu	Thr	Phe	Ser	Thr	Gly	Trp	Gly	Gln
	9	8	7	6	5	4	3	2	1
y''	-	882.411	781.363	634.295	547.263	446.215	389.194	203.114	146.093
y~	-	864.400	763.353	616.284	529.252	428.205	371.183	185.104	128.082
y*	-	865.384	764.337	617.268	530.236	429.189	372.167	186.088	129.066

Figure S15. MS/MS spectra of the singly- and the doubly-charged ions of the peptide detected at *m/z* 1106.53 assigned to sequence pQLTFSTGWGQa for Ostnu-AKH in *O. nubilalis* and the calculation of the expected fragment ions using MassLynx (blocked termini: pQ and amidation).

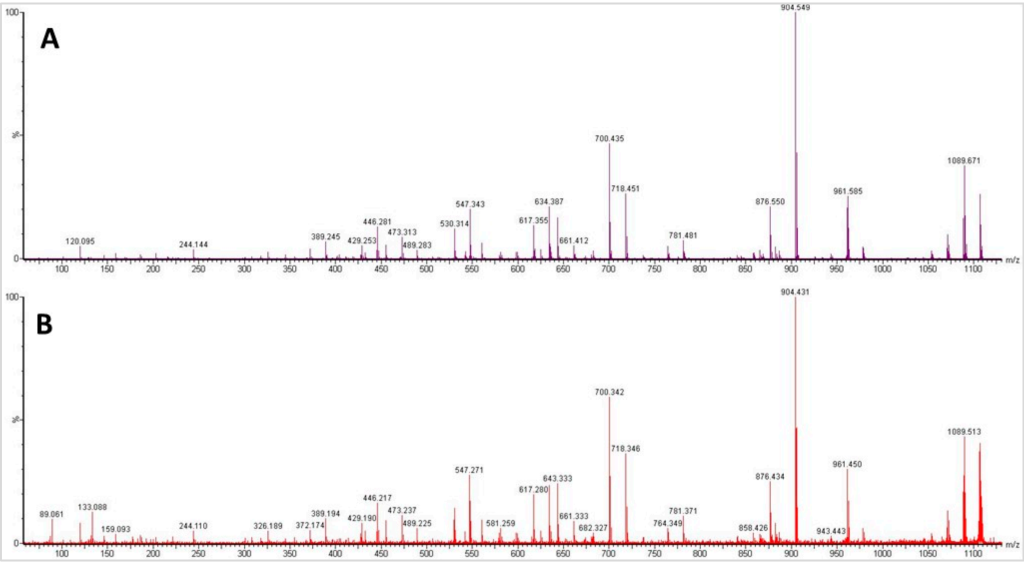

Figure S16. MS/MS spectra of the singly- and the doubly-charged ions of the peptide detected at *m/z* 1106.53 assigned to sequence pQLTFSTGWGQa for Ostnu-AKH in *O. nubilalis*. A) Standard peptide (34.2 min) and B) CC extract (34.3) were run with the same MS/MS method.

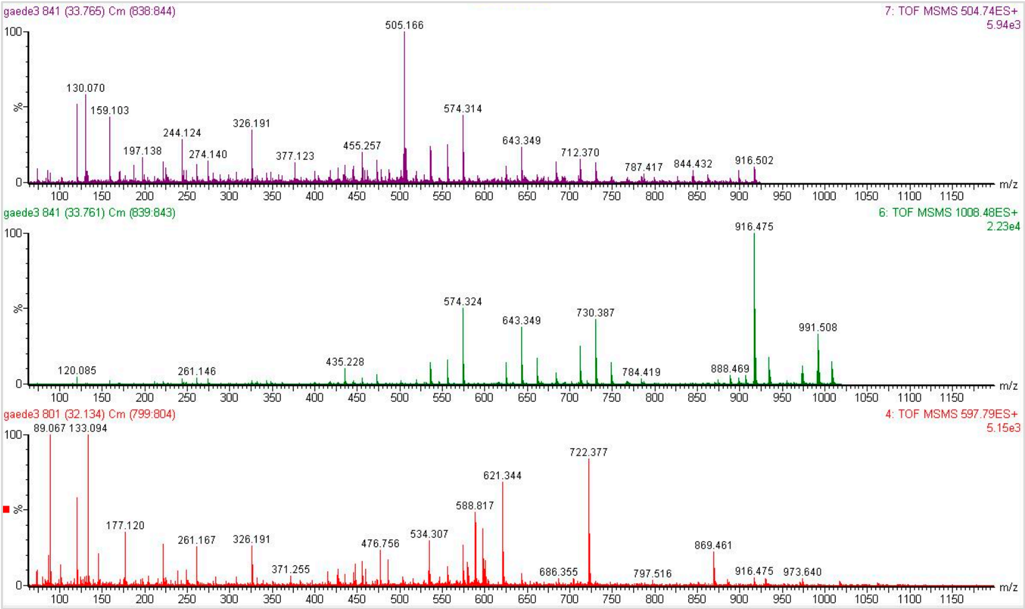

Figure S17. MS/MS spectra of the singly- and the doubly-charged ions of the peptides detected for Manse- and Vanca-AKH in *C. culmella.*

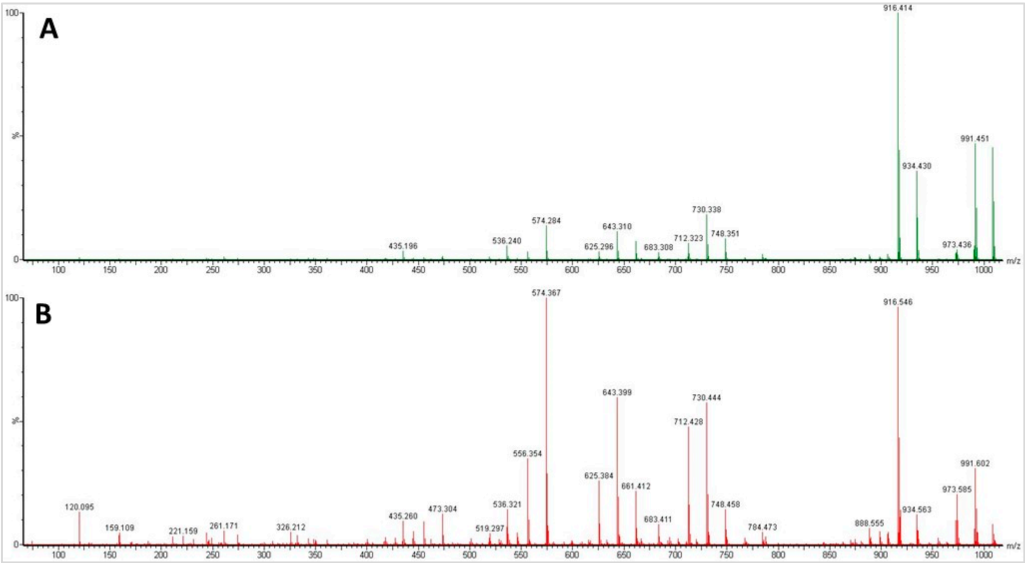

Figure S18. MS/MS spectra of the singly- and the doubly-charged ions of the peptides detected for Manse-AKH in *C. culmella.* A) Standard peptide and B) CC extract were run with slightly different collision energy (15-20 eV and 17-26 eV), but the fragments are identical.

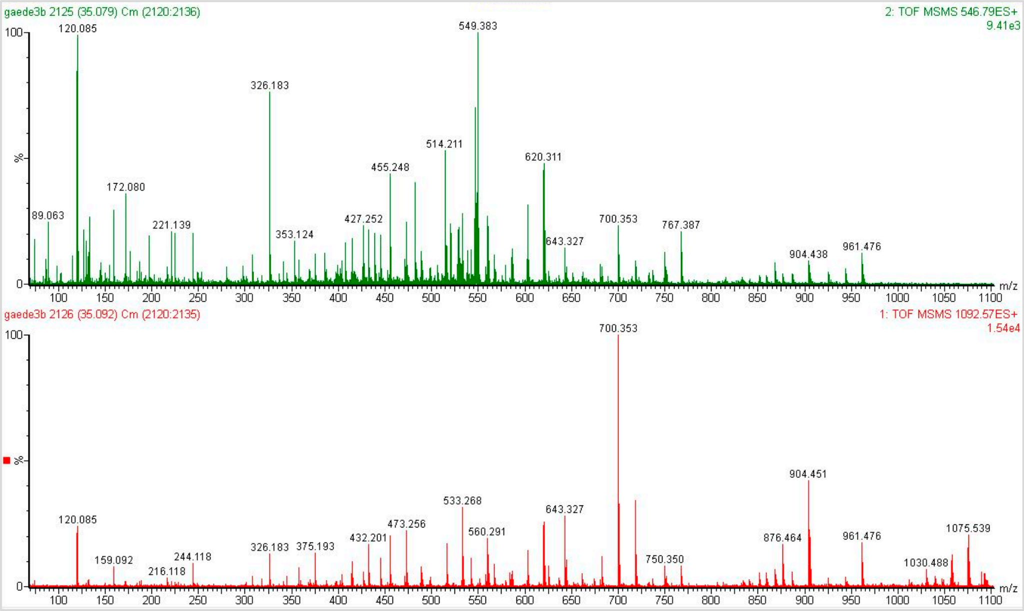

**b**	225.124	326.172	473.240	560.272	661.320	718.341	904.421	961.442	-
**i**	86.097	74.061	120.081	60.045	74.061	30.034	159.092	30.034	87.056
**b~**	207.113	308.161	455.229	542.262	643.309	700.331	886.410	943.431	-
**b***	208.097	309.145	456.214	543.246	644.293	701.315	887.394	944.415	-
	1	2	3	4	5	6	7	8	9
	**Leu**	**Thr**	**Phe**	**Ser**	**Thr**	**Gly**	**Trp**	**Gly**	**Asn**
	9	8	7	6	5	4	3	2	1
**y''**	-	868.395	767.348	620.279	533.247	432.200	375.178	189.099	132.077
**y~**	-	850.385	749.337	602.269	515.237	414.189	357.168	171.088	114.067
**y***	-	851.369	750.321	603.253	516.221	415.173	358.152	172.072	115.051

Figure S19. MS/MS spectra of the singly- and the doubly-charged ions of the peptide detected at *m/z* 1092.57 assigned to sequence pQLTFSTGWGNa for Chipa-AKH in *C. culmella* and the calculation of the expected fragment ions using MassLynx (blocked termini: pQ and amidation).

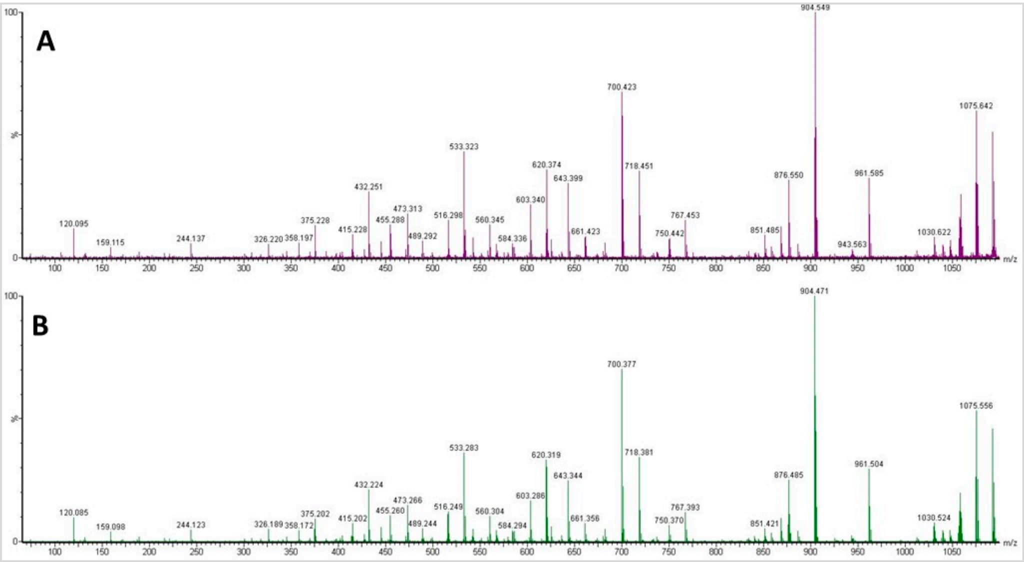

Figure S20. MS/MS spectra of the singly-charged ions of the peptide detected at *m/z* 1092.57 assigned to sequence pQLTFSTGWGNa for Chipa-AKH in *C. culmella.* A) CC extract and B) standard peptide were analyzed with the same MS/MS method.

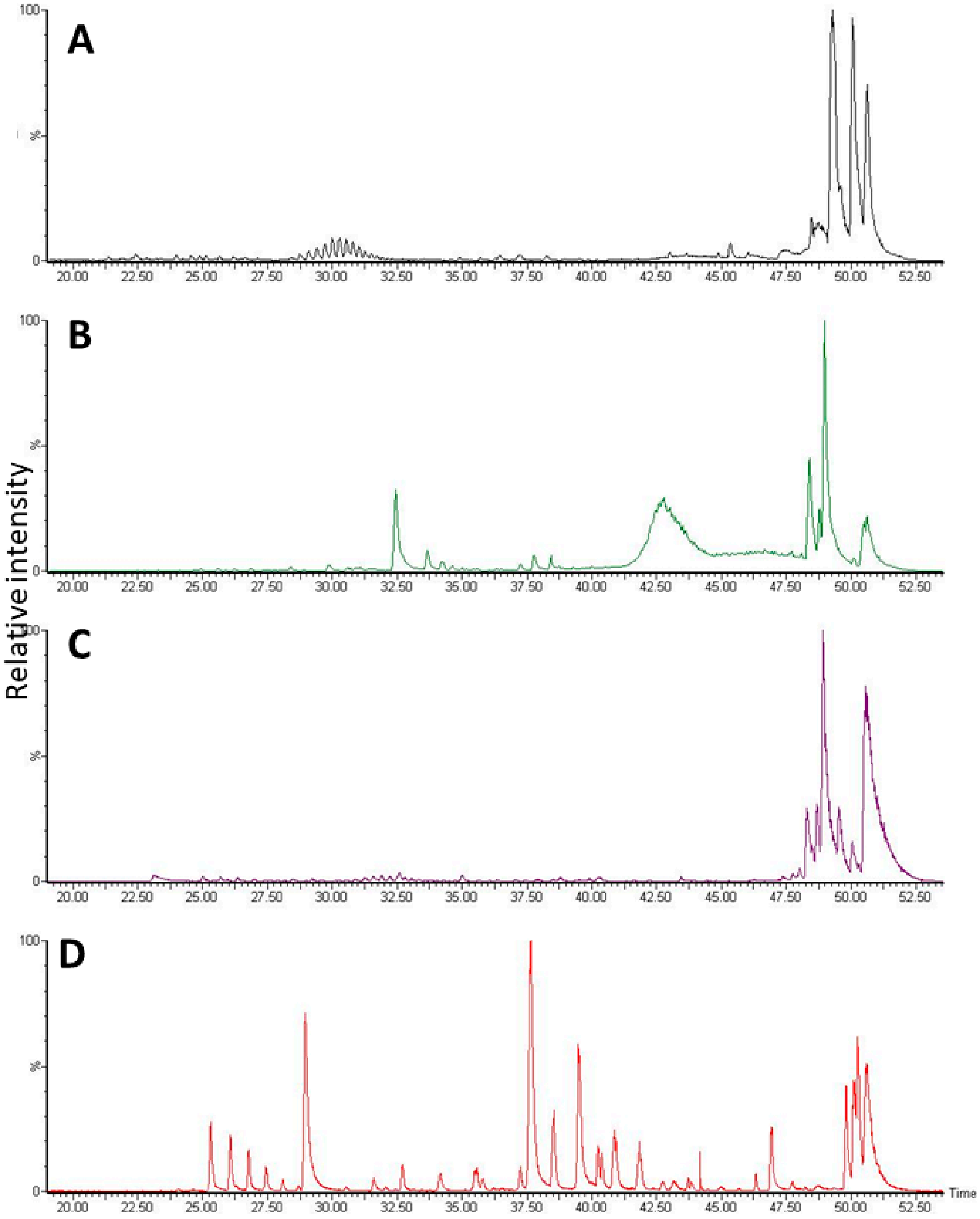

Figure S21. LC-chromatograms for the base peak in the overview scans for the CC extract of A) *P. tapulus*, B) *C. culmella* C) *O. nubilalis,* D) *K. flavicollis.*
